# Increasing accuracy of HLA imputation by a population-specific reference panel in a FinnGen biobank cohort

**DOI:** 10.1093/nargab/lqaa030

**Published:** 2020-05-06

**Authors:** Jarmo Ritari, Kati Hyvärinen, Jonna Clancy, FinnGen, Jukka Partanen, Satu Koskela

**Affiliations:** Research and Development, Finnish Red Cross Blood Service, Kivihaantie 7, 00310 Helsinki, Finland

## Abstract

The HLA genes, the most polymorphic genes in the human genome, constitute the strongest single genetic susceptibility factor for autoimmune diseases, transplantation alloimmunity and infections. HLA imputation via statistical inference of alleles based on single-nucleotide polymorphisms (SNPs) in linkage disequilibrium (LD) with alleles is a powerful first-step screening tool. Due to different LD structures between populations, the accuracy of HLA imputation may benefit from matching the imputation reference with the study population. To evaluate the potential advantage of using population-specific reference in HLA imputation, we constructed an HLA reference panel consisting of 1150 Finns with 5365 major histocompatibility complex region SNPs consistent between genome builds. We evaluated the accuracy of the panel against a European panel in an independent test set of 213 Finnish subjects. We show that the Finnish panel yields a lower imputation error rate (1.24% versus 1.79%). More than 30% of imputation errors occurred in haplotypes enriched in Finland. The frequencies of imputed HLA alleles were highly correlated with clinical-grade HLA allele frequencies and allowed accurate replication of established HLA–disease associations in ∼102 000 biobank participants. The results show that a population-specific reference increases imputation accuracy in a relatively isolated population within Europe and can be successfully applied to biobank-scale genome data collections.

## INTRODUCTION

The major histocompatibility complex (MHC) is regarded as a super-locus for genes playing central roles in the initiation and regulation of the immune system (1). The number of HLA alleles has exploded during the past 5–10 years because of advanced DNA sequencing techniques; today >20 000 alleles are described in the IMGT/HLA database (2).

Even though the high-resolution determination of HLA alleles for transplantation purposes is becoming more cost-effective and fast, in particular, due to NGS sequencing techniques, there is still a need for alternative, fast and low-cost screening of HLA alleles from large genome-wide association study cohorts. Computational methods have been created for predicting HLA alleles from single-nucleotide polymorphism (SNP) markers (3–5). By combining these resources with adequate computational power, a reliable screening of HLA alleles from large study cohorts is now feasible (6).

HLA imputation benefits from high linkage disequilibrium (LD) in the MHC region; therefore, despite immense variation, a relatively small number of SNPs provide a fairly good basis for allele prediction. Even so, imputation accuracy is highly dependent on the algorithm, reference population and coverage of the MHC region (7–11). Careful planning of an imputation pipeline and a reference panel are essential for high-accuracy HLA imputation. An accurate HLA imputation tool enables systematic studies of the landscape of MHC–disease associations and the discovery of novel associations in large population cohorts of hundreds of thousands of participants (12,13).

The Finnish population is a compilation of a few small founder populations with subsequent bottlenecks and scarce gene flow from Western and Eastern European populations shaping it as a genetic outlier in Europe (14–17). Over the course of history, the population was further divided into subisolates creating a genetic patchwork in Finland (18). These events may explain the specific HLA landscape with a reduced allele pool (19,20) and enrichment of particular HLA alleles and haplotypes in the current Finnish population (21,22). More than 30 HLA haplotypes that have been found to be common in Finland but rare or missing in other populations have been identified and named as Finnish enriched rare (FER) haplotypes (22).

In the present study, we describe the construction and validation of a high-accuracy HLA imputation panel for the Finnish population. We (i) collected a Finnish reference dataset and trained the HIBAG imputation program on it; (ii) compared the impact of different imputation algorithms and reference panels on imputation accuracy; and (iii) validated the Finnish reference panel by comparing the results with HLA allele frequencies in clinical-grade datasets and HLA–disease associations in over 100 000 genotypes of the FinnGen study cohort.

## MATERIALS AND METHODS

### Ethical permits

This study was carried out in accordance with the recommendations of the Ethical Review Board of the Hospital District of Helsinki and Uusimaa (decisions HUS/382/13/03/01/2014 and HUS/990/2017). A written informed or a broad biobank research consent was obtained for all living study participants. The National Supervisory Authority for Welfare and Health, Valvira, approved the study for deceased subjects (Dnro V/74832/2017).

## SAMPLE COLLECTION AND GENOTYPING

### Study cohorts

#### Reference dataset

The clinical HLA typing of the reference dataset of 1150 independent Finnish samples was performed by the HLA Laboratory of the Finnish Red Cross Blood Service using procedures accredited by the European Federation for Immunogenetics. Allele assignment of the seven classical HLA genes at two-field resolution level (i.e. unique protein sequence level) was performed by any of the three polymerase chain reaction (PCR) -based methods: sequence specific oligonucleotide probes (SSOP), sequence specific primers (SSP) or sequence based typing (SBT), as described in our previous study (23). SNP data of the reference dataset of 1150 Finnish individuals with pre-existing clinical HLA assignment were produced with Illumina Global Screening Array 24 v2 with Multi-disease drop-in (GSA, Illumina, Inc.) by the Institute for Molecular Medicine Finland, University of Helsinki. The numbers of different alleles of the HLA-A HLA-B, HLA-C, HLA-DRB1, HLA-DQA1, HLA-DQB1 and HLA-DPB1 genes in the reference dataset were 27, 40, 23, 33, 15, 16 and 24, respectively (Supplementary Table S1).

#### Test dataset

The SNP data of 426 study subjects in the test set were acquired by short-read sequencing of full MHC region acquired as described in (24), and are essentially identical to dataset described in (23). As the subjects were HLA-matched sibling pairs, they effectively constituted 213 independent samples. HLA typing at two-field resolution level for the seven classical HLA genes HLA-A, HLA-B, HLA-C, HLA-DRB1, HLA-DQA1, HLA-DQB1 and HLA-DPB1 (2982 HLA allele assignments) was performed by using Omixon Explore software v1.2.0 with IMGT 3.25.0_3 HLA database and by manual inspection of read alignments when necessary (23).

#### Validation dataset

An independent dataset of Finnish subjects with pre-existing HLA allele assignment was collected for validation of the imputation panel (i.e. the reference dataset). Factual allele frequencies of the seven classical HLA genes were acquired either from the Finnish Stem Cell Registry member database or from the Blood Service clinical laboratory database (Finnish subjects *n* = 21 068–26 329). HLA-A, HLA-B, HLA-C, HLA-DRB1 and HLA-DPB1 allele assignments were produced by Histogenetics Inc., USA. Due to technical challenges in short-read alignment with some alleles in HLA-DQA1 and HLA-DQB1 genes, only historical allele assignments produced by reference methods by the Finnish Tissue typing laboratory were qualified (*n* = 810 and 884, respectively). Imputed HLA allele frequencies of the target dataset were compared to the validation dataset allele frequencies.

#### Target dataset

The FinnGen research project aims to recruit biobank consents and DNA samples from 500 000 Finns through Finnish biobanks for systemic genotype–phenotype analyses (https://www.finngen.fi/en/Forresearchers). The members of the FinnGen consortium are listed in the Supplementary Data. FinnGen data freeze R2 consists of the genotype and phenotype data from 102 739 Finnish individuals (25). The genotyping procedure is described in (26).

### Imputation of HLA frequencies

**Figure 1.**
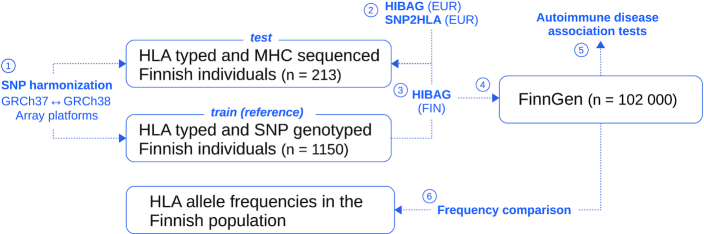
Schematic diagram of the analysis steps. (1) MHC SNPs were selected for consistency between array platforms and genome builds. The SNP genotype data with clinical-grade HLA types were divided into training and test sets. (2) HIBAG and SNP2HLA programs with their default references were applied to the test set to compare their accuracies. (3) HIBAG models were built on the training set and applied to the test set to analyze the impact of population-specific reference data on imputation accuracy. (4) HIBAG models trained on the Finnish reference data were applied to FinnGen R2 cohort. (5) The imputed HLA were tested for possible associations with six common autoimmune disorders to replicate known risk alleles. (6) Frequencies of the imputed HLAs were compared with frequencies from an independent clinical-grade HLA dataset.

The outline of the analysis pipeline is presented in Figure 1.

To harmonize the sets of MHC region SNPs between the Genome Reference Consortium Human Build 37 (GRCh37) and GRCh38, and the array platforms used in genotyping the reference data and FinnGen biobank data, the SNP lists from the different platforms were first intersected and thereafter queried against the ENSEMBL databases by their rs-IDs using R v3.5 and the library biomaRt v2.38.0 (27,28). SNPs that preserved their alleles in both genome builds were accepted for downstream analyses.

Two commonly used SNP-based stand-alone HLA imputation methods with pre-built European reference panel, SNP2HLA v1.0.3 (29) and HIBAG v1.14.0 (30), were applied to the test data. The imputation results were compared with the sequence-based and manually examined HLA typing results to calculate error rates for the seven HLA genes.

HIBAG models with 100 classifiers for each of the seven HLA genes were fitted using the training data of 1150 individuals to construct an imputation reference for the Finnish population. The reference was then applied to the test set to calculate imputation error rates and to compare with the results obtained using the European reference set. The HIBAG European ancestry reference set has been built on the 1958 British birth cohort, Wellcome Trust Case Control Consortium (http://www.biostat.washington.edu/∼bsweir/HIBAG/). In all imputations with HIBAG, variant positions in a corresponding genome build were used to select the SNPs from the target data.

The FinnGen R2 cohort subjects were HLA imputed using HIBAG with the Finnish HLA reference for HLA-A, HLA-B, HLA-C, HLA-DRB1 and HLA-DPB1, and the European reference for HLA-DQA1 and HLA-DQB1. The cohort was split into 10 subsets of equal size (10 200 each) and each subset was imputed separately. The imputed allele frequencies in each batch were compared with clinical-grade HLA typing reference frequencies. To analyze the effect of the HIBAG posterior probability on the frequency of imputed versus reference allele, we performed the frequency comparison at varying posterior probability cutoffs.

The imputed HLA types in FinnGen R2 were used to carry out association analyses against six common autoimmune disorders with established HLA risk alleles: type 1 diabetes (T1D; 61 225 controls and 348 cases), celiac disease (69 073 controls and 535 cases), psoriatic arthritis (50 119 controls and 344 cases), psoriasis (69 309 controls and 1115 cases), rheumatoid arthritis (58 385 controls and 1189 cases) and multiple sclerosis (31 971 controls and 230 cases). The analysis was performed using logistic regression of HLA allele dosage against case–control status, and using the 10 first genetic principal components, age, sex and BMI as covariates. After adjusting for the number of tested alleles and diseases, a *P*-value of 5e−5 was considered significant. To analyze the effect of the HIBAG posterior probability on the effect size of the main risk HLA allele, we performed the association testing at varying posterior probability cutoff values.

## RESULTS

### Overall imputation accuracy

First, we compared the SNP2HLA and HIBAG programs using their pre-built European reference datasets. HIBAG performed better than SNP2HLA for the HLA-A, HLA-B, HLA-C, HLA-DQA1 and HLA-DRB1 genes as measured by overall accuracy (95–98% and 82–98%, respectively), whereas SNP2HLA was better for the HLA-DQB1 gene (data not shown). Further imputations were performed by using HIBAG due to its better performance compared to the other algorithms also in previous studies (7,11,31). The outline of the imputation is shown in Figure 1.

The set of MHC region SNPs that were available on the array platforms and that were consistent between genome builds comprised altogether 5365 SNPs. The number of SNPs used by the fitted models was 4866.

**Figure 2.**
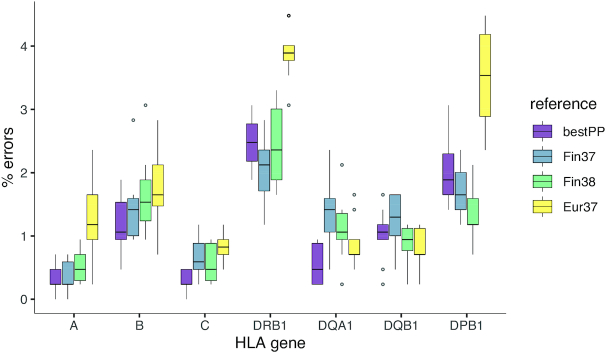
Impact of HIBAG reference data on imputation error. HLA genes are on the *x*-axis and the *y*-axis shows the number of imputation errors based on the test data (*n* = 213). The box plots (Tukey) show the error distributions from 100 bootstraps of the test data. ‘Fin37’ and ‘Fin38’ denote the Finnish reference on genome builds GRCh37 and GRCh38, respectively, and ‘Eur37’ denotes the European reference on GRCh37 (i.e. the HIBAG default built on the 1958 British birth cohort, Wellcome Trust Case Control Consortium). ‘bestPP’ indicates the selection of the best imputation reference according to HIBAG posterior probability.

Imputation accuracy in terms of the frequency of erroneously imputed alleles in each HLA gene was evaluated by applying the trained HIBAG models on an independent test dataset of 213 individuals for whom HLA genotypes were done by short-read paired-end sequencing. The number of errors varied modestly between loci and remained within a median range of 0.2–3.9% (Figure 2). The Finnish reference (Fin37) data yielded lower error rates than the default European reference (Eur37) for HLA-A, HLA-B, HLA-C, HLA-DRB1 and HLA-DPB1 genes, while the Eur37 yielded a lower error rate for HLA-DQA1 and HLA-DQB1 genes (Figure 2, Supplementary Table S2). The differences were largest for HLA-DRB1 (median [interquartile range, IQR] (%), 2.1 [0.6] for Fin37 versus 3.9 [0.2] for Eur37) and HLA-DPB1 (1.7 [0.6] for Fin37 versus 3.5 [1.3] for Eur37). Allele-specific errors between the three genome builds are shown in Supplementary File 2. Expectedly, the differences in error rate between the Finnish reference in GRCh37 and GRCh38 coordinates were small as these were based on the same SNPs. To evaluate whether the error rate was affected by selecting the best reference according to the imputation posterior probability, 10-fold cross-validation was used to select the reference with the highest probability in nine data subsets and calculate its error in the remaining subset systematically through all 10 subsets. The error rate of the probability-based selection was not superior relative to the Finnish reference, even though it yielded the best estimate for HLA-B, HLA-C and HLA-DQA1 genes (Figure 2).

**Figure 3.**
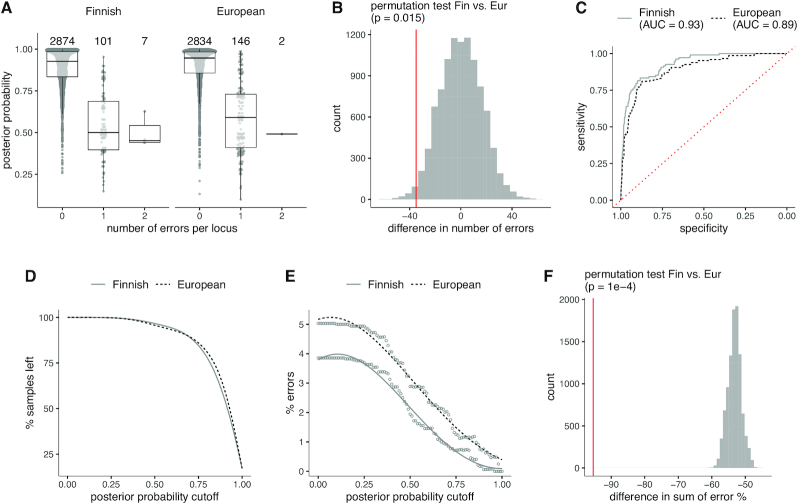
Imputation accuracy relative to HIBAG posterior probability. (**A**) HIBAG posterior probability distributions by the number of imputation errors per gene (*x*-axis) for the Finnish and European reference panels. (**B**) Statistical permutation test for the difference in total number of errors between the Finnish and European reference panels. (**C**) AUC/ROC analysis of imputation accuracy using posterior probabilities. One or two errors were grouped into a single category and analyzed against zero errors. (**D**) Proportion of retained samples by posterior probability threshold. A sample is discarded if its probability falls below a threshold value (*x*-axis). (**E**) Imputation error rate over the posterior probability threshold values (*x*-axis) for the Finnish and European reference panels. (**F**) Statistical permutation test for the difference in total error rate. The analyzed quantity is the sum of error rates over the probability threshold values. In all analyses, both the Finnish and European reference panels are in GRCh37/hg19 genome build coordinates.

The median level (IQR) of posterior probability rendering zero erroneously imputed alleles was 0.93 (0.15) for the Fin37 reference and 0.95 (0.12) for the Eur37 reference (Figure 3A). The total number of errors per locus statistically significantly differed between Fin37 and Eur37 (108 versus 148, respectively; *P* = 0.015) (Figure 3A and B). The diagnostic accuracy measured by the receiver operating characteristic (ROC) area under the curve (AUC) was 0.93 for Fin37 and 0.89 for Eur37 (Figure 3C). The references exhibited no difference in the proportion of discarded samples over the range of probability cutoff values (Figure 3D). Additionally, the differences between error proportions at varied probability cutoffs significantly differed between Fin37 and Eur37 (*P* = 1 × 10^−4^) (Figure 3E and F). The posterior probabilities of imputed HLA alleles are listed in Supplementary Table S3.

### Sample-specific errors

The number and distribution of imputation errors per sample with the two reference panels of different sizes and ethnic origins were investigated. More samples with imputation error were observed with the pre-built European than with the Finnish panel, 117 (28%) and 76 (18%), respectively (Supplementary Table S4). The number of errors per sample was, however, higher with the Finnish reference panel; individual samples could carry up to four errors with the European reference but up to six errors with the Finnish reference. Based on the known HLA haplotype frequencies, the errors seemed to originate from single and specific haplotypes, an observation noticeable especially with the Finnish reference panel; there were at most three errors per putative HLA haplotype with the European reference but as many as six errors with the Finnish one. We also evaluated the proportion of imputation errors that originated from the FER HLA haplotypes (22). Altogether 32% of the errors were found in samples with FER haplotypes with the European reference panel, while the proportion was 11% with the Finnish reference.

### Frequencies of imputed HLA alleles in FinnGen R2 cohort

**Figure 4.**
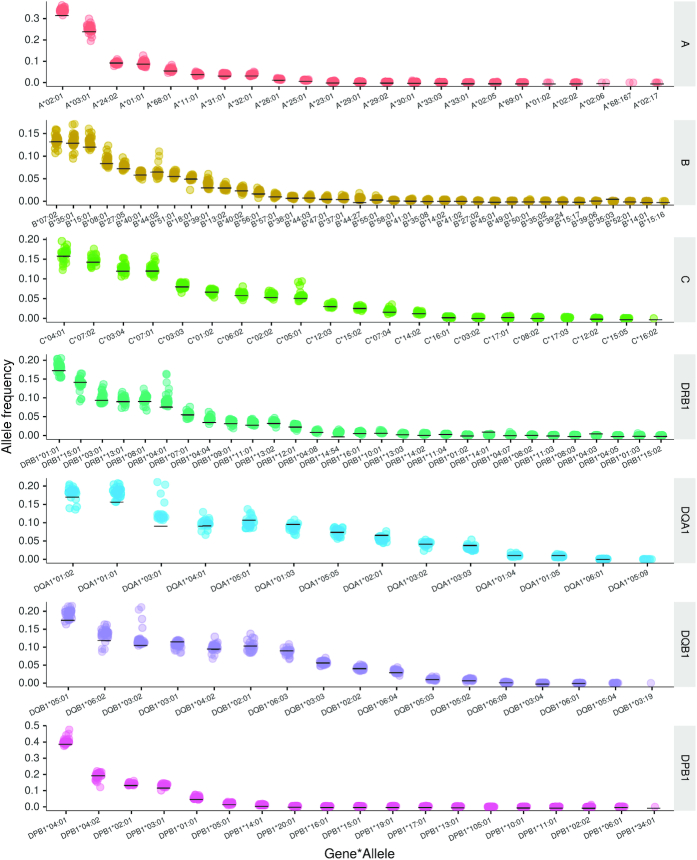
Congruency of the imputed HLA allele frequencies and the clinical-grade HLA frequencies. Imputed HLA allele frequencies from the FinnGen R2 genotyping batches (total subjects *n* ∼ 102 000) in comparison with frequencies from an independent, clinical-grade HLA-typed Finnish dataset (*n* ∼ 25 000). The horizontal black bars indicate the population frequencies from the independent dataset. Each colored data point represents FinnGen R2 genotyping batch (the total number of genotyping batches is 32).

HLA imputation was performed separately for the FinnGen R2 genotype batches (*n* = 32). Figure 4 presents the frequencies of imputed HLA alleles and the corresponding levels in an independent cohort of HLA frequencies, obtained by clinical-grade HLA typing. The mean value with standard deviation for each allele is listed in Supplementary Table S5. The mean differences between imputed allele frequencies and the independent dataset with clinical HLA type varied from −1.3% to +3.0%. The largest variation was observed among DQA1*03:01 (3.0 ± 2.6%), DQA1*01:01 (2.3 ± 1.3%), A*02:01 (1.9 ± 0.9%) and DRB1*04:01 (1.8 ± 2.2%). For all other alleles, the modulus of mean difference was ≤1.5%. The observed variation was related to the size of the subcohort and thereby to the number of imputed alleles. There was a negative correlation between the mean frequency deviation and the number of imputed alleles (Supplementary Figure S1). The imputed versus reference allele frequencies at varying posterior probability cutoffs are presented in Supplementary Figure S2.

### Association of imputed HLA alleles with six common autoimmune diseases

**Figure 5.**
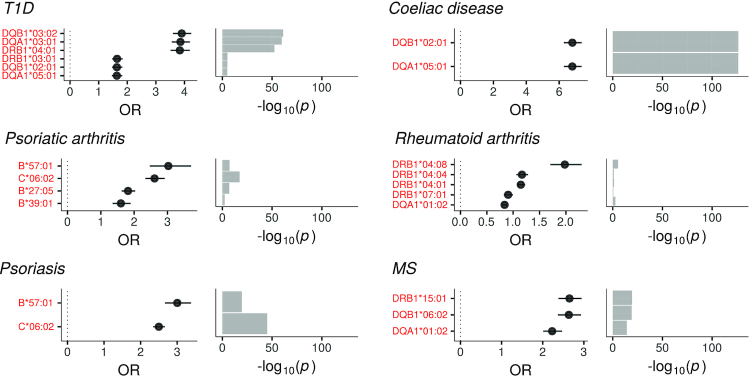
Imputed HLA alleles and their associations with autoimmune disorders in the FinnGen R2 cohort. Previously established risk alleles for six autoimmune disorders emerged in the association analyses. The test statistic is log odds ratio from logistic regression analysis using age, sex, BMI and 10 genetic principal components as covariates. The horizontal bars in the left-hand side panels indicate 95% confidence intervals for the test statistic. T1D = type 1 diabetes; MS = multiple sclerosis.

HLA alleles are known to be strong risk factors for many immunological diseases, such as celiac disease, T1D, psoriasis, psoriatic arthritis, rheumatoid arthritis and multiple sclerosis. To further validate the imputation accuracy and usefulness in large biobank-scale cohorts, these previously published HLA associations were evaluated using the imputed HLA alleles and clinical data of the FinnGen R2 cohort. As demonstrated in Figure 5, all established top associations could be confirmed. The effect sizes are listed in Supplementary Table S6. The observed effect directions correlated with previously published results derived from Global Biobank Engine (https://biobankengine.stanford.edu/hla-assoc). The autoimmune disorder associations at varying HIBAG posterior probability cutoffs for the main risk HLA allele are presented in Supplementary Figure S3.

## DISCUSSION

In the present study, we have evaluated the impact of a population-specific HLA reference panel on the imputation accuracy of seven classical HLA genes at two-field resolution level (i.e. unique protein sequence) in the Finns. We focused on HLA imputation from SNP markers with the purpose of application to large biobank collections containing SNP-based genotype data.

The results obtained from the comparison of Finnish and European reference panels underline the importance of matching the reference population with the target population, suggesting that the LD structure and allele frequencies differing even between genetically relatively close populations, such as the Central Europeans and the Finns, are significant for imputation performance. While previous studies have shown the importance of building specific reference panels for non-European populations (8), our results show that this principle applies also within the Europe. In addition to the Finnish population, also other genetically isolated European populations, such as Sardinians or Basques, might benefit from population-specific imputation panels.

Although some of the imputation errors are explained simply by uncertainty arising from low allele frequencies, errors made by the European reference were concentrated particularly on haplotypes enriched in the Finnish population (FER) (22). More than 30% of the imputation errors with the European reference panel occurred in samples with at least one FER, suggesting that the European panel was not able to fully capture the target population LD structure despite containing informative SNP markers. This kind of landscape of HLA haplotypes is in line with other genetic studies on Finnish genetic structure (15,16,18). The particular genetic structure has proved to be useful for mapping single-gene defects and could be exploited in studying HLA genetics of complex diseases as well. The high LD in HLA haplotypes has been a hurdle for fine-mapping HLA-linked risk variants, but may be overcome by cross-population comparisons of the same trait to single out the shared associated variant, emphasizing the need for accurate HLA imputation of not only FERs but haplotypes specific to other populations as well.

The rates of false allele imputation of HLA-A, HLA-B, HLA-C, HLA-DQA1 and HLA-DQB1 genes were relatively low—at the level of 1–2%—regardless of the reference population used. As array genotyping is inexpensive as compared to clinical HLA typing, imputation based on SNP data provides an excellent tool for HLA association screening. However, using the European reference panel produced incorrectly imputed HLA-DRB1 and HLA-DPB1 alleles at the rate of ∼4%, which was twice the rate of the Finnish panel. Such error rates may be acceptable for a general first-step association but may lead to inaccurate results in fine mappings or small strictly defined subpopulations. For example, accurate imputation of the DRB1*04 group is needed in diabetes studies as the alleles of the DRB1*04 group differ in their risk effect (32). As associations with the HLA-DPB1 gene have not been studied as intensively as other HLA genes, accurate imputation of HLA-DPB1 alleles with a population-specific reference panel may turn to be valuable. Certainly, imputation could be a fast way to increase the number of HLA-typed blood donors whose platelet units can be used for leukemia patients in need of HLA-typed platelets.

We applied the HLA imputation panel to a large biobank genotype data collection. The FinnGen data freeze R2 included over 100 000 genotypes from Finnish individuals for whom we had no prior HLA data. First, we could demonstrate that the frequencies of imputed HLA alleles in the FinnGen material were concordant with the frequencies of an independent dataset of ∼25 000 potential stem cell donors with clinical HLA type. The majority of the FinnGen material originates from the Finnish hospital biobanks and disease-specific study cohorts. Importantly, the overall HLA allele frequencies were in line with those assumed, but there were certain batches with biased frequencies suggesting that those may include high numbers of a specific patient group; the increased frequencies of HLA-DRB1*04:01, HLA-DQA1*03:01 and HLA-DQB1*03:02 alleles in a few batches are most likely due to a high number of T1D patients in these batches. Second, we could validate the HLA imputation procedure by testing whether we can find the well-established HLA autoimmune disease associations in the FinnGen phenotype groups. Clearly, the top associations in the FinnGen material were those assumed based on earlier reports. Also, the effect sizes of the associations were consistent with those reported, testifying for the accuracy and practical utility of the imputation panel. Nevertheless, as the FinnGen material consists of samples from hospital biobank participants, there might have been some bias due to, for example, control frequencies.

An obvious limitation of the present study is that the method is able to impute only HLA types present in the reference dataset. Additionally, the posterior probability of imputation could be influenced by allele frequency as rare alleles are imputed with lower certainty. According to our results, removal of alleles with probability <0.5 does not affect frequency or association estimates markedly, but it should be emphasized that for rare alleles this threshold may be too stringent. Ideally, the reference data should be comprehensive in terms of allele representation to ensure high-accuracy HLA imputation. For example, in this study, reinforcing the reference dataset with a larger Finnish cohort together with samples from the neighbouring populations could improve the imputation accuracy as the Finnish genome is an admixture of these (17,33–35). The number and coverage of HLA panels is expected to increase as multi-ethnic datasets are already being collected by global collaboration (36) and workshops (the SNP-HLA Reference Consortium, https://www.ihiw18.org/component-bio-informatics/snp-hla-reference/).

In summary, by forming a population-specific reference panel and its application to the FinnGen biobank cohort, we were able to impute HLA types for ∼2% of the Finnish population and demonstrate the value of population-matched reference for HLA analysis of large genome data cohorts. While our approach was limited by the relatively small number of individuals included in the panel, the panel was able to capture the LD structure of the target population, helping to improve the imputation accuracy of haplotypes where more general reference panels are prone to make errors. Thus, scrutinizing the associations of HLA variation with various phenotypes in rapidly growing biobank genome data collections from different populations is expected to benefit from specified reference panels.

## DATA AVAILABILITY

Imputation models, summary statistic data and R code are available at GitHub (https://github.com/FRCBS/HLA-imputation). The data do not contain any individual identifying information.

## Supplementary Material

lqaa030_Supplemental_Files
