# Supplementary material for: Increasing accuracy of HLA imputation by a population-specific reference panel in a FinnGen biobank cohort
Source: NAR Genom Bioinform. 2020 May 6;2(2):lqaa030. doi: 10.1093/nargab/lqaa030 (PMC7671345; doi:10.1093/nargab/lqaa030)
Supplement: lqaa030_Supplemental_Files [file lqaa030_supplemental_files.zip › HLA imputation_Supplementary Data_ NARgb_revision.docx]

**SUPPLEMENTARY DATA**

Increasing accuracy of HLA imputation by a population-specific reference panel in a FinnGen biobank cohort

Jarmo Ritari^1^, Kati Hyvärinen^1^, Jonna Clancy^1^, FinnGen, Jukka Partanen^1^, Satu Koskela^1^

^1^ Finnish Red Cross Blood Service, Helsinki, Finland

Number of Supplementary Tables 6

Number of Supplementary Figures 3

**Supplementary Tables**

**Supplementary Table 1. Number of HLA alleles in the Finnish reference data set (n = 1150).**

| **HLA-A** | | **HLA-B** | | **HLA-C** | | **HLA-DRB1** | | **HLA-DQA1** | | **HLA-DQB1** | | | **HLA-DPB1** | |
| --- | --- | --- | --- | --- | --- | --- | --- | --- | --- | --- | --- | --- | --- | --- |
| **allele** | **n** | **allele** | **n** | **allele** | **n** | **allele** | **n** | **allele** | **n** | **allele** | **n** | | **allele** | **n** |
| A*01:01 | 202 | B*07:01 | 1 | C*01:02 | 151 | DRB1*01:01 | 471 | DQA1*01:01 | 389 | DQB1*02:01 | 235 | | DPB1*01:01 | 122 |
| A*01:02 | 1 | B*07:02 | 334 | C*02:02 | 116 | DRB1*01:02 | 6 | DQA1*01:02 | 338 | DQB1*02:02 | 78 | | DPB1*02:01 | 285 |
| A*02:01 | 701 | B*08:01 | 199 | C*03:02 | 7 | DRB1*01:03 | 5 | DQA1*01:03 | 168 | DQB1*03:01 | 223 | | DPB1*02:02 | 5 |
| A*02:02 | 1 | B*13:01 | 1 | C*03:03 | 158 | DRB1*03:01 | 234 | DQA1*01:04 | 28 | DQB1*03:02 | 216 | | DPB1*03:01 | 287 |
| A*02:03 | 1 | B*13:02 | 47 | C*03:04 | 242 | DRB1*04:01 | 179 | DQA1*01:05 | 15 | DQB1*03:03 | 117 | | DPB1*04:01 | 934 |
| A*02:05 | 2 | B*14:01 | 1 | C*04:01 | 368 | DRB1*04:02 | 2 | DQA1*02:01 | 93 | DQB1*03:04 | 2 | | DPB1*04:02 | 395 |
| A*02:06 | 1 | B*14:02 | 10 | C*04:06 | 1 | DRB1*04:03 | 8 | DQA1*03:01 | 146 | DQB1*03:05 | 1 | | DPB1*05:01 | 56 |
| A*02:07 | 1 | B*15:01 | 237 | C*05:01 | 81 | DRB1*04:04 | 63 | DQA1*03:02 | 79 | DQB1*04:02 | 202 | | DPB1*06:01 | 8 |
| A*02:17 | 1 | B*15:16 | 1 | C*06:02 | 103 | DRB1*04:05 | 3 | DQA1*03:03 | 27 | DQB1*05:01 | 502 | | DPB1*09:01 | 3 |
| A*03:01 | 559 | B*15:17 | 2 | C*07:01 | 254 | DRB1*04:07 | 3 | DQA1*04:01 | 166 | DQB1*05:02 | 31 | | DPB1*10:01 | 4 |
| A*11:01 | 65 | B*18:01 | 85 | C*07:02 | 345 | DRB1*04:08 | 18 | DQA1*04:02 | 1 | DQB1*05:03 | 33 | | DPB1*11:01 | 3 |
| A*23:01 | 17 | B*27:02 | 4 | C*07:04 | 43 | DRB1*07:01 | 110 | DQA1*05:01 | 207 | DQB1*06:01 | 3 | | DPB1*13:01 | 14 |
| A*24:02 | 132 | B*27:05 | 143 | C*08:02 | 9 | DRB1*07:03 | 1 | DQA1*05:03 | 9 | DQB1*06:02 | 344 | | DPB1*14:01 | 28 |
| A*25:01 | 25 | B*35:01 | 312 | C*12:02 | 2 | DRB1*08:01 | 200 | DQA1*05:05 | 116 | DQB1*06:03 | 221 | | DPB1*15:01 | 7 |
| A*26:01 | 26 | B*35:02 | 4 | C*12:03 | 62 | DRB1*08:02 | 5 | DQA1*06:01 | 3 | DQB1*06:04 | 54 | | DPB1*16:01 | 14 |
| A*29:01 | 6 | B*35:03 | 4 | C*14:02 | 39 | DRB1*08:03 | 3 |  |  | DQB1*06:09 | 5 | | DPB1*17:01 | 10 |
| A*29:02 | 7 | B*35:08 | 3 | C*15:02 | 47 | DRB1*09:01 | 67 |  |  |  |  | | DPB1*19:01 | 11 |
| A*30:01 | 2 | B*37:01 | 13 | C*15:05 | 1 | DRB1*10:01 | 16 |  |  |  |  | | DPB1*20:01 | 13 |
| A*30:02 | 1 | B*38:01 | 12 | C*16:01 | 6 | DRB1*11:01 | 74 |  |  |  |  | | DPB1*23:01 | 6 |
| A*31:01 | 63 | B*39:01 | 52 | C*16:02 | 2 | DRB1*11:03 | 3 |  |  |  |  | | DPB1*25:01 | 1 |
| A*32:01 | 59 | B*39:06 | 2 | C*17:01 | 6 | DRB1*11:04 | 14 |  |  |  |  | | DPB1*31:01 | 1 |
| A*33:01 | 5 | B*39:24 | 2 | C*17:03 | 3 | DRB1*12:01 | 48 |  |  |  |  | | DPB1*34:01 | 1 |
| A*33:03 | 5 | B*40:01 | 110 | C*03:327 | 1 | DRB1*13:01 | 234 |  |  |  |  | | DPB1*105:01 | 3 |
| A*33:05 | 1 | B*40:02 | 38 |  |  | DRB1*13:02 | 60 |  |  |  | |  |  |  |
| A*68:01 | 93 | B*41:01 | 6 |  |  | DRB1*13:03 | 8 |  |  |  | |  |  |  |
| A*69:01 | 2 | B*41:02 | 3 |  |  | DRB1*13:05 | 1 |  |  |  | |  |  |  |
| A*68:167 | 1 | B*44:02 | 95 |  |  | DRB1*13:32 | 1 |  |  |  | |  |  |  |
|  |  | B*44:03 | 23 |  |  | DRB1*14:01 | 11 |  |  |  | |  |  |  |
|  |  | B*44:27 | 22 |  |  | DRB1*14:02 | 10 |  |  |  | |  |  |  |
|  |  | B*45:01 | 3 |  |  | DRB1*14:54 | 19 |  |  |  | |  |  |  |
|  |  | B*46:01 | 1 |  |  | DRB1*15:01 | 342 |  |  |  | |  |  |  |
|  |  | B*47:01 | 8 |  |  | DRB1*15:02 | 5 |  |  |  | |  |  |  |
|  |  | B*49:01 | 5 |  |  | DRB1*16:01 | 24 |  |  |  | |  |  |  |
|  |  | B*50:01 | 1 |  |  |  |  |  |  |  | |  |  |  |
|  |  | B*51:01 | 107 |  |  |  |  |  |  |  | |  |  |  |
|  |  | B*52:01 | 3 |  |  |  |  |  |  |  | |  |  |  |
|  |  | B*55:01 | 13 |  |  |  |  |  |  |  | |  |  |  |
|  |  | B*56:01 | 54 |  |  |  |  |  |  |  | |  |  |  |
|  |  | B*57:01 | 23 |  |  |  |  |  |  |  | |  |  |  |
|  |  | B*58:01 | 8 |  |  |  |  |  |  |  | |  |  |  |

**Supplementary Table 2. Proportion of imputation errors using different references.**

| **Reference** | **HLA gene** | **Median (%)** | **Interquartile range (%)** |
| --- | --- | --- | --- |
| bestPP | A | 0.2 | 0.2 |
| bestPP | B | 1.1 | 0.6 |
| bestPP | C | 0.2 | 0.2 |
| bestPP | DRB1 | 2.5 | 0.6 |
| bestPP | DQA1 | 0.5 | 0.6 |
| bestPP | DQB1 | 1.1 | 0.2 |
| bestPP | DPB1 | 1.9 | 0.6 |
| Fin37 | A | 0.2 | 0.4 |
| Fin37 | B | 1.4 | 0.6 |
| Fin37 | C | 0.6 | 0.4 |
| Fin37 | DRB1 | 2.1 | 0.6 |
| Fin37 | DQA1 | 1.4 | 0.5 |
| Fin37 | DQB1 | 1.3 | 0.6 |
| Fin37 | DPB1 | 1.7 | 0.6 |
| Fin38 | A | 0.5 | 0.4 |
| Fin38 | B | 1.5 | 0.6 |
| Fin38 | C | 0.5 | 0.6 |
| Fin38 | DRB1 | 2.4 | 1.1 |
| Fin38 | DQA1 | 1.1 | 0.4 |
| Fin38 | DQB1 | 0.9 | 0.4 |
| Fin38 | DPB1 | 1.2 | 0.4 |
| Eur37 | A | 1.2 | 0.7 |
| Eur37 | B | 1.7 | 0.6 |
| Eur37 | C | 0.8 | 0.2 |
| Eur37 | DRB1 | 3.9 | 0.2 |
| Eur37 | DQA1 | 0.7 | 0.2 |
| Eur37 | DQB1 | 0.7 | 0.4 |
| Eur37 | DPB1 | 3.5 | 1.3 |

bestPP. best posterior probability reference; Eur37. the European reference in Genome Reference Consortium Human Build 37; Fin37. Finnish reference in Genome Reference Consortium Human Build 37; Fin38. Finnish reference in Genome Reference Consortium Human Build 38.

**Supplementary Table 3. Posterior probabilities of imputed HLA alleles**

| **Locus** | **Allele** | **n** | **Posterior probability**  **(mean)** | **Standard deviation** | **Posterior probability**  **(median)** | **Interquartile range** |
| --- | --- | --- | --- | --- | --- | --- |
| A | 03:01 | 43707 | 0.802 | 0.089 | 0.813 | 0.103 |
| A | 01:01 | 16913 | 0.830 | 0.099 | 0.849 | 0.105 |
| A | 02:01 | 56007 | 0.801 | 0.096 | 0.813 | 0.108 |
| A | 26:01 | 3384 | 0.794 | 0.117 | 0.821 | 0.142 |
| A | 32:01 | 7284 | 0.830 | 0.096 | 0.844 | 0.111 |
| A | 31:01 | 7674 | 0.830 | 0.121 | 0.861 | 0.101 |
| A | 11:01 | 8043 | 0.803 | 0.102 | 0.823 | 0.105 |
| A | 24:02 | 17688 | 0.772 | 0.110 | 0.789 | 0.137 |
| A | 68:01 | 11605 | 0.839 | 0.096 | 0.854 | 0.100 |
| A | 25:01 | 2339 | 0.834 | 0.122 | 0.865 | 0.130 |
| A | 29:01 | 647 | 0.796 | 0.209 | 0.880 | 0.164 |
| A | 30:01 | 536 | 0.658 | 0.131 | 0.701 | 0.190 |
| A | 23:01 | 708 | 0.820 | 0.129 | 0.851 | 0.127 |
| A | 02:05 | 155 | 0.506 | 0.116 | 0.548 | 0.102 |
| A | 29:02 | 599 | 0.818 | 0.198 | 0.886 | 0.131 |
| A | 02:02 | 36 | 0.338 | 0.031 | 0.332 | 0.039 |
| A | 33:03 | 369 | 0.824 | 0.100 | 0.846 | 0.096 |
| A | 33:01 | 174 | 0.757 | 0.153 | 0.790 | 0.153 |
| A | 02:06 | 2 | 0.478 | 0.004 | 0.478 | 0.003 |
| A | 02:17 | 5 | 0.388 | 0.006 | 0.392 | 0.004 |
| A | 01:02 | 4 | 0.454 | 0.027 | 0.451 | 0.025 |
| A | 69:01 | 139 | 0.612 | 0.098 | 0.637 | 0.098 |
| A | 68:167 | 2 | 0.420 | 0.065 | 0.420 | 0.046 |
| B | 07:02 | 24640 | 0.789 | 0.108 | 0.816 | 0.097 |
| B | 35:01 | 24747 | 0.818 | 0.114 | 0.847 | 0.103 |
| B | 08:01 | 17036 | 0.843 | 0.111 | 0.870 | 0.099 |
| B | 15:01 | 24354 | 0.768 | 0.120 | 0.789 | 0.133 |
| B | 18:01 | 9888 | 0.787 | 0.137 | 0.819 | 0.183 |
| B | 27:05 | 14939 | 0.801 | 0.119 | 0.830 | 0.128 |
| B | 13:02 | 5834 | 0.865 | 0.115 | 0.896 | 0.090 |
| B | 44:02 | 12118 | 0.759 | 0.131 | 0.777 | 0.149 |
| B | 40:01 | 11864 | 0.810 | 0.113 | 0.836 | 0.108 |
| B | 41:01 | 607 | 0.808 | 0.219 | 0.894 | 0.169 |
| B | 47:01 | 1445 | 0.827 | 0.140 | 0.863 | 0.132 |
| B | 38:01 | 1755 | 0.750 | 0.143 | 0.787 | 0.165 |
| B | 40:02 | 5166 | 0.799 | 0.133 | 0.830 | 0.137 |
| B | 39:01 | 7591 | 0.811 | 0.154 | 0.864 | 0.174 |
| B | 56:01 | 3911 | 0.868 | 0.118 | 0.898 | 0.103 |
| B | 35:08 | 480 | 0.511 | 0.128 | 0.533 | 0.160 |
| B | 44:27 | 1485 | 0.860 | 0.118 | 0.888 | 0.107 |
| B | 15:17 | 132 | 0.547 | 0.089 | 0.568 | 0.093 |
| B | 51:01 | 11638 | 0.753 | 0.168 | 0.802 | 0.219 |
| B | 14:02 | 522 | 0.806 | 0.186 | 0.876 | 0.174 |
| B | 41:02 | 404 | 0.796 | 0.162 | 0.848 | 0.136 |
| B | 57:01 | 2391 | 0.854 | 0.136 | 0.889 | 0.096 |
| B | 37:01 | 1536 | 0.799 | 0.131 | 0.836 | 0.116 |
| B | 35:03 | 154 | 0.522 | 0.149 | 0.514 | 0.137 |
| B | 44:03 | 1654 | 0.835 | 0.165 | 0.887 | 0.114 |
| B | 35:02 | 167 | 0.442 | 0.092 | 0.466 | 0.064 |
| B | 39:24 | 210 | 0.458 | 0.062 | 0.463 | 0.074 |
| B | 45:01 | 206 | 0.652 | 0.179 | 0.684 | 0.193 |
| B | 14:01 | 134 | 0.482 | 0.073 | 0.491 | 0.081 |
| B | 49:01 | 228 | 0.710 | 0.176 | 0.732 | 0.179 |
| B | 27:02 | 214 | 0.621 | 0.130 | 0.632 | 0.203 |
| B | 39:06 | 138 | 0.687 | 0.118 | 0.715 | 0.120 |
| B | 55:01 | 1014 | 0.730 | 0.158 | 0.775 | 0.166 |
| B | 50:01 | 206 | 0.457 | 0.147 | 0.446 | 0.254 |
| B | 58:01 | 568 | 0.747 | 0.196 | 0.838 | 0.337 |
| B | 52:01 | 139 | 0.423 | 0.124 | 0.425 | 0.210 |
| B | 15:16 | 6 | 0.484 | 0.055 | 0.475 | 0.065 |
| C | 04:01 | 29324 | 0.862 | 0.099 | 0.888 | 0.103 |
| C | 01:02 | 13170 | 0.903 | 0.106 | 0.933 | 0.085 |
| C | 02:02 | 11209 | 0.889 | 0.105 | 0.916 | 0.089 |
| C | 07:01 | 23411 | 0.885 | 0.094 | 0.908 | 0.069 |
| C | 03:04 | 23853 | 0.873 | 0.113 | 0.903 | 0.099 |
| C | 07:02 | 27115 | 0.882 | 0.092 | 0.902 | 0.077 |
| C | 06:02 | 11617 | 0.902 | 0.098 | 0.929 | 0.080 |
| C | 03:03 | 15991 | 0.887 | 0.123 | 0.923 | 0.098 |
| C | 05:01 | 10998 | 0.817 | 0.132 | 0.846 | 0.119 |
| C | 07:04 | 3784 | 0.889 | 0.130 | 0.932 | 0.099 |
| C | 12:03 | 6745 | 0.909 | 0.112 | 0.942 | 0.073 |
| C | 03:02 | 592 | 0.781 | 0.185 | 0.863 | 0.276 |
| C | 14:02 | 2790 | 0.905 | 0.132 | 0.947 | 0.078 |
| C | 15:02 | 5385 | 0.872 | 0.141 | 0.920 | 0.109 |
| C | 08:02 | 538 | 0.768 | 0.203 | 0.859 | 0.345 |
| C | 17:01 | 598 | 0.845 | 0.222 | 0.939 | 0.115 |
| C | 16:01 | 725 | 0.833 | 0.150 | 0.868 | 0.198 |
| C | 12:02 | 153 | 0.265 | 0.079 | 0.254 | 0.127 |
| C | 17:03 | 396 | 0.826 | 0.135 | 0.877 | 0.100 |
| C | 15:05 | 50 | 0.635 | 0.066 | 0.658 | 0.047 |
| C | 16:02 | 1 | 0.325 | NA | 0.325 | 0.000 |
| DRB1 | 01:01 | 32914 | 0.929 | 0.125 | 0.980 | 0.063 |
| DRB1 | 03:01 | 18847 | 0.930 | 0.137 | 0.990 | 0.056 |
| DRB1 | 13:01 | 17797 | 0.906 | 0.149 | 0.966 | 0.089 |
| DRB1 | 04:01 | 18135 | 0.863 | 0.184 | 0.951 | 0.174 |
| DRB1 | 08:01 | 18185 | 0.930 | 0.132 | 0.984 | 0.057 |
| DRB1 | 15:01 | 25170 | 0.931 | 0.129 | 0.983 | 0.051 |
| DRB1 | 07:01 | 10778 | 0.922 | 0.148 | 0.987 | 0.069 |
| DRB1 | 09:01 | 7025 | 0.918 | 0.142 | 0.976 | 0.077 |
| DRB1 | 04:04 | 8852 | 0.820 | 0.199 | 0.914 | 0.291 |
| DRB1 | 04:08 | 2289 | 0.885 | 0.169 | 0.963 | 0.118 |
| DRB1 | 14:54 | 1917 | 0.655 | 0.213 | 0.680 | 0.375 |
| DRB1 | 11:01 | 6988 | 0.821 | 0.178 | 0.880 | 0.209 |
| DRB1 | 11:03 | 168 | 0.558 | 0.097 | 0.593 | 0.111 |
| DRB1 | 13:02 | 6873 | 0.901 | 0.153 | 0.973 | 0.126 |
| DRB1 | 10:01 | 1764 | 0.893 | 0.166 | 0.962 | 0.106 |
| DRB1 | 12:01 | 5022 | 0.904 | 0.143 | 0.952 | 0.070 |
| DRB1 | 01:03 | 89 | 0.726 | 0.176 | 0.727 | 0.278 |
| DRB1 | 14:01 | 429 | 0.547 | 0.190 | 0.521 | 0.331 |
| DRB1 | 04:07 | 324 | 0.594 | 0.158 | 0.660 | 0.294 |
| DRB1 | 01:02 | 384 | 0.837 | 0.173 | 0.914 | 0.173 |
| DRB1 | 04:03 | 107 | 0.628 | 0.150 | 0.671 | 0.261 |
| DRB1 | 14:02 | 792 | 0.922 | 0.136 | 0.979 | 0.074 |
| DRB1 | 13:03 | 938 | 0.922 | 0.143 | 0.981 | 0.065 |
| DRB1 | 15:02 | 111 | 0.743 | 0.204 | 0.823 | 0.307 |
| DRB1 | 11:04 | 700 | 0.603 | 0.138 | 0.611 | 0.159 |
| DRB1 | 08:02 | 272 | 0.591 | 0.109 | 0.555 | 0.175 |
| DRB1 | 08:03 | 199 | 0.607 | 0.192 | 0.639 | 0.369 |
| DRB1 | 16:01 | 1735 | 0.856 | 0.194 | 0.947 | 0.164 |
| DRB1 | 04:05 | 68 | 0.563 | 0.084 | 0.594 | 0.072 |
| DQA1 | 01:01 | 33358 | 0.933 | 0.095 | 0.975 | 0.084 |
| DQA1 | 05:01 | 18767 | 0.935 | 0.099 | 0.978 | 0.083 |
| DQA1 | 01:02 | 32514 | 0.912 | 0.113 | 0.957 | 0.108 |
| DQA1 | 03:01 | 23236 | 0.802 | 0.123 | 0.849 | 0.146 |
| DQA1 | 01:03 | 16492 | 0.895 | 0.109 | 0.938 | 0.102 |
| DQA1 | 04:01 | 18477 | 0.916 | 0.104 | 0.965 | 0.100 |
| DQA1 | 01:05 | 1699 | 0.648 | 0.103 | 0.668 | 0.148 |
| DQA1 | 02:01 | 10817 | 0.927 | 0.105 | 0.971 | 0.086 |
| DQA1 | 01:04 | 2266 | 0.825 | 0.120 | 0.866 | 0.095 |
| DQA1 | 03:03 | 6017 | 0.798 | 0.120 | 0.841 | 0.095 |
| DQA1 | 05:05 | 14652 | 0.898 | 0.132 | 0.954 | 0.125 |
| DQA1 | 03:02 | 8265 | 0.779 | 0.128 | 0.804 | 0.167 |
| DQA1 | 06:01 | 206 | 0.833 | 0.124 | 0.877 | 0.151 |
| DQA1 | 05:09 | 24 | 0.635 | 0.148 | 0.661 | 0.263 |
| DQB1 | 05:01 | 34809 | 0.961 | 0.065 | 0.982 | 0.043 |
| DQB1 | 03:01 | 20386 | 0.909 | 0.110 | 0.939 | 0.091 |
| DQB1 | 02:01 | 18760 | 0.956 | 0.071 | 0.979 | 0.045 |
| DQB1 | 05:02 | 1953 | 0.881 | 0.106 | 0.920 | 0.060 |
| DQB1 | 06:02 | 24794 | 0.929 | 0.084 | 0.956 | 0.066 |
| DQB1 | 04:02 | 18634 | 0.951 | 0.080 | 0.976 | 0.054 |
| DQB1 | 02:02 | 8020 | 0.950 | 0.072 | 0.976 | 0.054 |
| DQB1 | 03:03 | 10980 | 0.856 | 0.119 | 0.883 | 0.135 |
| DQB1 | 03:02 | 23083 | 0.928 | 0.069 | 0.948 | 0.050 |
| DQB1 | 05:03 | 2273 | 0.929 | 0.122 | 0.970 | 0.055 |
| DQB1 | 06:03 | 16431 | 0.908 | 0.093 | 0.937 | 0.074 |
| DQB1 | 06:04 | 6501 | 0.850 | 0.114 | 0.907 | 0.141 |
| DQB1 | 03:04 | 106 | 0.600 | 0.066 | 0.620 | 0.061 |
| DQB1 | 06:01 | 113 | 0.914 | 0.127 | 0.961 | 0.074 |
| DQB1 | 05:04 | 37 | 0.548 | 0.065 | 0.552 | 0.091 |
| DQB1 | 03:19 | 1 | 0.499 | NA | 0.499 | 0.000 |
| DQB1 | 06:09 | 399 | 0.897 | 0.099 | 0.934 | 0.083 |
| DPB1 | 02:01 | 25956 | 0.934 | 0.101 | 0.968 | 0.055 |
| DPB1 | 01:01 | 11020 | 0.950 | 0.104 | 0.985 | 0.042 |
| DPB1 | 04:01 | 64085 | 0.935 | 0.105 | 0.973 | 0.060 |
| DPB1 | 03:01 | 25148 | 0.923 | 0.126 | 0.971 | 0.065 |
| DPB1 | 04:02 | 34433 | 0.923 | 0.096 | 0.959 | 0.063 |
| DPB1 | 05:01 | 4599 | 0.936 | 0.105 | 0.974 | 0.060 |
| DPB1 | 02:02 | 211 | 0.695 | 0.129 | 0.724 | 0.190 |
| DPB1 | 13:01 | 660 | 0.684 | 0.208 | 0.751 | 0.368 |
| DPB1 | 11:01 | 336 | 0.638 | 0.147 | 0.688 | 0.221 |
| DPB1 | 10:01 | 354 | 0.591 | 0.155 | 0.566 | 0.160 |
| DPB1 | 14:01 | 2612 | 0.785 | 0.193 | 0.827 | 0.317 |
| DPB1 | 15:01 | 1011 | 0.787 | 0.132 | 0.785 | 0.154 |
| DPB1 | 16:01 | 984 | 0.894 | 0.122 | 0.935 | 0.122 |
| DPB1 | 19:01 | 943 | 0.917 | 0.101 | 0.945 | 0.066 |
| DPB1 | 105:01 | 359 | 0.699 | 0.093 | 0.721 | 0.083 |
| DPB1 | 17:01 | 845 | 0.768 | 0.148 | 0.783 | 0.232 |
| DPB1 | 20:01 | 1029 | 0.672 | 0.172 | 0.663 | 0.249 |
| DPB1 | 06:01 | 105 | 0.472 | 0.077 | 0.491 | 0.093 |
| DPB1 | 34:01 | 1 | 0.232 | NA | 0.232 | 0.000 |

NA, not available

| **Supplementary Table 4. Imputation errors in the HLA-A, -B, -C, -DRB1, -DQA1, -DQB1 (and -DPB1) genes** | | | | |  |  |
| --- | --- | --- | --- | --- | --- | --- |
|  |  |  |  |  |  |  |
|  |  |  |  |  |  |  |
| **Samples (n=426)** | **EUR37** | | | **FIN37** | | |
| 7 HLA genes | **samples (n)** | **samples (%)** | **errors (%)** | **samples (n)** | **samples (%)** | **errors (%)** |
| **all imputation errors** | **117** | **0.275** | **1.000** | **76** | **0.178** | **1.000** |
| 1 error/sample | 91 | 0.214 | 0.778 | 56 | 0.131 | 0.737 |
| 2 errors/sample | 18 | 0.042 | 0.154 | 7 | 0.016 | 0.092 |
| 3 errors/sample | 7 | 0.016 | 0.060 | 6 | 0.014 | 0.079 |
| 4 errors/sample | 1 | 0.002 | 0.009 | 3 | 0.007 | 0.039 |
| 5 errors/sample | 0 | 0 | 0.000 | 2 | 0.005 | 0.026 |
| 6 errors/sample | 0 | 0 | 0.000 | 2 | 0.005 | 0.026 |
|  |  |  |  |  |  |  |
|  |  |  |  |  |  |  |
| **Haplotypes* (n=852)** | **EUR37** | | | **FIN37** | | |
| errors in 6 HLA genes | **samples (n)** | **samples (%)** | **errors (%)** | **samples (n)** | **samples (%)** | **errors (%)** |
| **all imputation errors** | **113** | **0.133** | **1.000** | **103** | **0.121** | **1.000** |
| 1 error/haplo | 73 | 0.086 | 0.820 | 45 | 0.053 | 0.714 |
| 2 errors/haplo | 8 | 0.009 | 0.090 | 6 | 0.007 | 0.095 |
| 3 errors/haplo | 8 | 0.009 | 0.090 | 6 | 0.007 | 0.095 |
| 4 errors/haplo | 0 | 0.000 | 0.000 | 4 | 0.005 | 0.063 |
| 5 errors/haplo | 0 | 0.000 | 0.000 | 0 | 0.000 | 0.000 |
| 6 errors/haplo | 0 | 0.000 | 0.000 | 2 | 0.002 | 0.032 |
| **errors in FERs**** | **36** | **0.042** | **0.319** | **11** | **0.013** | **0.107** |
| FER with 1 error | 28 | 0.033 | 0.875 | 7 | 0.008 | 0.778 |
| FER with 2 errors | 4 | 0.005 | 0.125 | 2 | 0.002 | 0.222 |
| **errors in non-FERs** | **77** | **0.090** | **0.681** | **92** | **0.108** | **0.893** |
| non-FER with 1 error | 45 | 0.053 | 0.789 | 38 | 0.045 | 0.679 |
| non-FER with 2 errors | 4 | 0.005 | 0.070 | 6 | 0.007 | 0.107 |
| non-FER with 3 errors | 8 | 0.009 | 0.140 | 6 | 0.007 | 0.107 |
| non-FER with 4 errors | 0 | 0.000 | 0.000 | 6 | 0.007 | 0.107 |
|  |  |  |  |  |  |  |

** Putative haplotypes based on the known HLA haplotype frequencies in NMDP and Finnish Stem Cell Registry*

*** FER motifs include*

**Supplementary Table 5. Frequencies of imputed HLA alleles**

| **Allele** | **Imputed allele frequency (mean)** | **Imputed allele frequency (SD)** | **Reference allele frequency** | | **Δ(allele frequency)^a^** | **Δ(allele frequency) SD** |
| --- | --- | --- | --- | --- | --- | --- |
| A*01:01 | 8.97E-02 | 1.16E-02 | | 9.30E-02 | -3.37E-03 | 1.16E-02 |
| A*01:02 | 2.79E-04 | 6.04E-05 | | 8.10E-05 | 1.98E-04 | 6.04E-05 |
| A*02:01 | 3.39E-01 | 9.01E-03 | | 3.20E-01 | 1.94E-02 | 9.01E-03 |
| A*02:02 | 2.37E-04 | 1.18E-04 | | 2.63E-04 | -2.61E-05 | 1.18E-04 |
| A*02:05 | 9.19E-04 | 9.39E-04 | | 1.03E-03 | -1.14E-04 | 9.39E-04 |
| A*02:06 | 1.94E-04 | 1.04E-04 | | 1.30E-03 | -1.10E-03 | 1.04E-04 |
| A*02:17 | 1.31E-04 | 1.71E-05 | | 2.02E-05 | 1.11E-04 | 1.71E-05 |
| A*03:01 | 2.52E-01 | 1.95E-02 | | 2.45E-01 | 6.88E-03 | 1.95E-02 |
| A*11:01 | 4.14E-02 | 4.91E-03 | | 4.50E-02 | -3.65E-03 | 4.91E-03 |
| A*23:01 | 3.70E-03 | 1.82E-03 | | 4.37E-03 | -6.73E-04 | 1.82E-03 |
| A*24:02 | 9.26E-02 | 5.87E-03 | | 9.81E-02 | -5.46E-03 | 5.87E-03 |
| A*25:01 | 1.16E-02 | 1.57E-03 | | 1.24E-02 | -8.14E-04 | 1.57E-03 |
| A*26:01 | 1.70E-02 | 2.38E-03 | | 1.83E-02 | -1.32E-03 | 2.38E-03 |
| A*29:01 | 3.31E-03 | 1.21E-03 | | 3.48E-03 | -1.73E-04 | 1.21E-03 |
| A*29:02 | 2.88E-03 | 7.87E-04 | | 3.97E-03 | -1.09E-03 | 7.87E-04 |
| A*30:01 | 2.76E-03 | 1.54E-03 | | 3.54E-03 | -7.87E-04 | 1.54E-03 |
| A*31:01 | 3.99E-02 | 3.82E-03 | | 3.78E-02 | 2.14E-03 | 3.82E-03 |
| A*32:01 | 3.84E-02 | 4.51E-03 | | 3.85E-02 | -1.15E-04 | 4.51E-03 |
| A*33:01 | 9.55E-04 | 4.80E-04 | | 1.05E-03 | -9.80E-05 | 4.80E-04 |
| A*33:03 | 1.85E-03 | 5.43E-04 | | 2.69E-03 | -8.40E-04 | 5.43E-04 |
| A*68:01 | 6.10E-02 | 6.90E-03 | | 6.17E-02 | -6.35E-04 | 6.90E-03 |
| A*68:167 | 1.31E-04 | 2.49E-05 | | NA | NA | NA |
| A*69:01 | 7.07E-04 | 4.26E-04 | | 5.47E-04 | 1.60E-04 | 4.26E-04 |
| B*07:02 | 1.36E-01 | 1.17E-02 | | 1.34E-01 | 1.20E-03 | 1.17E-02 |
| B*08:01 | 8.96E-02 | 1.11E-02 | | 8.66E-02 | 2.94E-03 | 1.11E-02 |
| B*13:02 | 2.96E-02 | 3.83E-03 | | 3.23E-02 | -2.65E-03 | 3.83E-03 |
| B*14:01 | 6.25E-04 | 3.00E-04 | | 5.43E-04 | 8.17E-05 | 3.00E-04 |
| B*14:02 | 2.49E-03 | 7.95E-04 | | 3.03E-03 | -5.41E-04 | 7.95E-04 |
| B*15:01 | 1.31E-01 | 1.09E-02 | | 1.23E-01 | 7.96E-03 | 1.09E-02 |
| B*15:16 | 3.02E-04 | 1.19E-04 | | 9.70E-05 | 2.05E-04 | 1.19E-04 |
| B*15:17 | 7.52E-04 | 4.12E-04 | | 9.90E-04 | -2.38E-04 | 4.12E-04 |
| B*18:01 | 5.05E-02 | 5.64E-03 | | 5.16E-02 | -1.19E-03 | 5.64E-03 |
| B*27:02 | 1.11E-03 | 5.69E-04 | | 1.77E-03 | -6.58E-04 | 5.69E-04 |
| B*27:05 | 7.88E-02 | 5.89E-03 | | 7.54E-02 | 3.47E-03 | 5.89E-03 |
| B*35:01 | 1.34E-01 | 1.60E-02 | | 1.32E-01 | 2.16E-03 | 1.60E-02 |
| B*35:02 | 1.02E-03 | 6.27E-04 | | 1.69E-03 | -6.67E-04 | 6.27E-04 |
| B*35:03 | 7.32E-04 | 3.12E-04 | | 7.28E-03 | -6.55E-03 | 3.12E-04 |
| B*35:08 | 2.55E-03 | 1.10E-03 | | 2.43E-03 | 1.22E-04 | 1.10E-03 |
| B*37:01 | 7.68E-03 | 2.26E-03 | | 6.93E-03 | 7.47E-04 | 2.26E-03 |
| B*38:01 | 8.72E-03 | 2.02E-03 | | 9.68E-03 | -9.65E-04 | 2.02E-03 |
| B*39:01 | 3.94E-02 | 7.25E-03 | | 3.25E-02 | 6.94E-03 | 7.25E-03 |
| B*39:06 | 8.37E-04 | 4.43E-04 | | 3.78E-03 | -2.95E-03 | 4.43E-04 |
| B*39:24 | 1.02E-03 | 5.96E-04 | | 1.55E-03 | -5.34E-04 | 5.96E-04 |
| B*40:01 | 6.11E-02 | 5.29E-03 | | 6.08E-02 | 2.87E-04 | 5.29E-03 |
| B*40:02 | 2.62E-02 | 4.31E-03 | | 2.63E-02 | -7.53E-05 | 4.31E-03 |
| B*41:01 | 2.96E-03 | 1.19E-03 | | 3.30E-03 | -3.36E-04 | 1.19E-03 |
| B*41:02 | 2.17E-03 | 6.56E-04 | | 2.79E-03 | -6.23E-04 | 6.56E-04 |
| B*44:02 | 6.45E-02 | 1.28E-02 | | 6.74E-02 | -2.90E-03 | 1.28E-02 |
| B*44:03 | 8.56E-03 | 2.24E-03 | | 9.96E-03 | -1.40E-03 | 2.24E-03 |
| B*44:27 | 7.55E-03 | 2.68E-03 | | 3.88E-05 | 7.51E-03 | 2.68E-03 |
| B*45:01 | 1.02E-03 | 4.39E-04 | | 1.11E-03 | -8.79E-05 | 4.39E-04 |
| B*47:01 | 7.65E-03 | 2.14E-03 | | 7.14E-03 | 5.08E-04 | 2.14E-03 |
| B*49:01 | 1.10E-03 | 4.50E-04 | | 1.71E-03 | -6.05E-04 | 4.50E-04 |
| B*50:01 | 1.18E-03 | 7.98E-04 | | 1.57E-03 | -3.95E-04 | 7.98E-04 |
| B*51:01 | 6.06E-02 | 4.91E-03 | | 5.75E-02 | 3.12E-03 | 4.91E-03 |
| B*52:01 | 6.02E-04 | 3.36E-04 | | 1.44E-03 | -8.34E-04 | 3.36E-04 |
| B*55:01 | 5.23E-03 | 2.00E-03 | | 5.80E-03 | -5.78E-04 | 2.00E-03 |
| B*56:01 | 2.00E-02 | 4.04E-03 | | 1.95E-02 | 4.85E-04 | 4.04E-03 |
| B*57:01 | 1.22E-02 | 2.75E-03 | | 1.29E-02 | -7.28E-04 | 2.75E-03 |
| B*58:01 | 2.76E-03 | 7.28E-04 | | 3.75E-03 | -9.85E-04 | 7.28E-04 |
| C*01:02 | 6.81E-02 | 4.90E-03 | | 7.03E-02 | -2.20E-03 | 4.90E-03 |
| C*02:02 | 5.88E-02 | 4.73E-03 | | 5.64E-02 | 2.42E-03 | 4.73E-03 |
| C*03:02 | 2.89E-03 | 7.31E-04 | | 3.60E-03 | -7.06E-04 | 7.31E-04 |
| C*03:03 | 8.35E-02 | 6.02E-03 | | 8.34E-02 | 4.07E-05 | 6.02E-03 |
| C*03:04 | 1.28E-01 | 1.10E-02 | | 1.23E-01 | 4.73E-03 | 1.10E-02 |
| C*04:01 | 1.60E-01 | 1.52E-02 | | 1.61E-01 | -9.12E-04 | 1.52E-02 |
| C*05:01 | 5.78E-02 | 1.16E-02 | | 5.44E-02 | 3.38E-03 | 1.16E-02 |
| C*06:02 | 6.03E-02 | 6.25E-03 | | 6.14E-02 | -1.10E-03 | 6.25E-03 |
| C*07:01 | 1.25E-01 | 1.16E-02 | | 1.23E-01 | 1.49E-03 | 1.16E-02 |
| C*07:02 | 1.49E-01 | 1.05E-02 | | 1.46E-01 | 3.59E-03 | 1.05E-02 |
| C*07:04 | 1.98E-02 | 4.42E-03 | | 1.91E-02 | 6.96E-04 | 4.42E-03 |
| C*08:02 | 2.57E-03 | 7.88E-04 | | 3.60E-03 | -1.03E-03 | 7.88E-04 |
| C*12:02 | 6.39E-04 | 4.06E-04 | | 1.44E-03 | -7.96E-04 | 4.06E-04 |
| C*12:03 | 3.39E-02 | 3.76E-03 | | 3.34E-02 | 5.14E-04 | 3.76E-03 |
| C*14:02 | 1.44E-02 | 2.37E-03 | | 1.51E-02 | -6.54E-04 | 2.37E-03 |
| C*15:02 | 2.70E-02 | 3.15E-03 | | 2.87E-02 | -1.73E-03 | 3.15E-03 |
| C*15:05 | 3.39E-04 | 1.82E-04 | | 6.68E-04 | -3.30E-04 | 1.82E-04 |
| C*16:01 | 3.65E-03 | 9.97E-04 | | 5.23E-03 | -1.58E-03 | 9.97E-04 |
| C*16:02 | 2.89E-04 | NA | | 1.97E-04 | 9.27E-05 | NA |
| C*17:01 | 2.91E-03 | 1.21E-03 | | 5.80E-03 | -2.89E-03 | 1.21E-03 |
| C*17:03 | 2.11E-03 | 7.07E-04 | | NA | NA | NA |
| DPB1*01:01 | 5.70E-02 | 7.05E-03 | | 5.53E-02 | 1.63E-03 | 7.05E-03 |
| DPB1*02:01 | 1.42E-01 | 6.34E-03 | | 1.41E-01 | 6.30E-04 | 6.34E-03 |
| DPB1*02:02 | 1.64E-03 | 2.76E-03 | | 9.02E-04 | 7.33E-04 | 2.76E-03 |
| DPB1*03:01 | 1.35E-01 | 8.47E-03 | | 1.25E-01 | 9.84E-03 | 8.47E-03 |
| DPB1*04:01 | 4.06E-01 | 1.99E-02 | | 3.94E-01 | 1.20E-02 | 1.99E-02 |
| DPB1*04:02 | 1.90E-01 | 2.04E-02 | | 2.01E-01 | -1.09E-02 | 2.04E-02 |
| DPB1*05:01 | 2.30E-02 | 4.16E-03 | | 2.34E-02 | -4.92E-04 | 4.16E-03 |
| DPB1*06:01 | 6.57E-04 | 4.11E-04 | | 4.75E-03 | -4.09E-03 | 4.11E-04 |
| DPB1*10:01 | 1.80E-03 | 7.24E-04 | | 2.30E-03 | -5.04E-04 | 7.24E-04 |
| DPB1*105:01 | 1.90E-03 | 7.68E-04 | | NA | NA | NA |
| DPB1*11:01 | 1.75E-03 | 6.64E-04 | | 2.06E-03 | -3.12E-04 | 6.64E-04 |
| DPB1*13:01 | 3.24E-03 | 1.08E-03 | | 3.77E-03 | -5.36E-04 | 1.08E-03 |
| DPB1*14:01 | 1.35E-02 | 2.50E-03 | | 1.22E-02 | 1.33E-03 | 2.50E-03 |
| DPB1*15:01 | 4.84E-03 | 1.32E-03 | | 5.08E-03 | -2.38E-04 | 1.32E-03 |
| DPB1*16:01 | 4.87E-03 | 8.94E-04 | | 5.27E-03 | -3.94E-04 | 8.94E-04 |
| DPB1*17:01 | 4.39E-03 | 1.09E-03 | | 5.06E-03 | -6.67E-04 | 1.09E-03 |
| DPB1*19:01 | 4.74E-03 | 1.38E-03 | | 5.15E-03 | -4.11E-04 | 1.38E-03 |
| DPB1*20:01 | 5.30E-03 | 1.37E-03 | | 7.36E-03 | -2.06E-03 | 1.37E-03 |
| DPB1*34:01 | 2.17E-04 | NA | | 9.49E-05 | 1.22E-04 | NA |
| DQA1*01:01 | 1.83E-01 | 1.27E-02 | | 1.60E-01 | 2.31E-02 | 1.27E-02 |
| DQA1*01:02 | 1.81E-01 | 1.55E-02 | | 1.74E-01 | 7.37E-03 | 1.55E-02 |
| DQA1*01:03 | 8.78E-02 | 8.47E-03 | | 9.86E-02 | -1.08E-02 | 8.47E-03 |
| DQA1*01:04 | 1.10E-02 | 2.39E-03 | | 1.43E-02 | -3.29E-03 | 2.39E-03 |
| DQA1*01:05 | 8.46E-03 | 2.11E-03 | | 1.43E-02 | -5.88E-03 | 2.11E-03 |
| DQA1*02:01 | 5.61E-02 | 6.54E-03 | | 6.90E-02 | -1.29E-02 | 6.54E-03 |
| DQA1*03:01 | 1.25E-01 | 2.57E-02 | | 9.41E-02 | 3.05E-02 | 2.57E-02 |
| DQA1*03:02 | 4.24E-02 | 5.47E-03 | | 4.48E-02 | -2.39E-03 | 5.47E-03 |
| DQA1*03:03 | 3.11E-02 | 5.92E-03 | | 4.12E-02 | -1.01E-02 | 5.92E-03 |
| DQA1*04:01 | 9.81E-02 | 1.11E-02 | | 9.41E-02 | 4.00E-03 | 1.11E-02 |
| DQA1*05:01 | 1.00E-01 | 1.33E-02 | | 1.10E-01 | -1.02E-02 | 1.33E-02 |
| DQA1*05:05 | 7.48E-02 | 8.67E-03 | | 7.71E-02 | -2.28E-03 | 8.67E-03 |
| DQA1*05:09 | 2.11E-04 | 9.20E-05 | | NA | NA | NA |
| DQA1*06:01 | 1.01E-03 | 5.98E-04 | | 3.58E-03 | -2.58E-03 | 5.98E-04 |
| DQB1*02:01 | 9.99E-02 | 1.33E-02 | | 1.07E-01 | -7.51E-03 | 1.33E-02 |
| DQB1*02:02 | 4.12E-02 | 4.52E-03 | | 4.44E-02 | -3.23E-03 | 4.52E-03 |
| DQB1*03:01 | 1.07E-01 | 9.49E-03 | | 1.20E-01 | -1.28E-02 | 9.49E-03 |
| DQB1*03:02 | 1.24E-01 | 2.60E-02 | | 1.09E-01 | 1.48E-02 | 2.60E-02 |
| DQB1*03:03 | 5.71E-02 | 4.74E-03 | | 5.98E-02 | -2.70E-03 | 4.74E-03 |
| DQB1*03:04 | 6.84E-04 | 4.34E-04 | | 8.08E-04 | -1.24E-04 | 4.34E-04 |
| DQB1*03:19 | 1.49E-04 | NA | | NA | NA | NA |
| DQB1*04:02 | 9.89E-02 | 1.09E-02 | | 9.85E-02 | 3.96E-04 | 1.09E-02 |
| DQB1*05:01 | 1.92E-01 | 1.22E-02 | | 1.79E-01 | 1.37E-02 | 1.22E-02 |
| DQB1*05:02 | 9.57E-03 | 2.13E-03 | | 1.05E-02 | -9.27E-04 | 2.13E-03 |
| DQB1*05:03 | 1.10E-02 | 2.39E-03 | | 1.37E-02 | -2.69E-03 | 2.39E-03 |
| DQB1*05:04 | 2.96E-04 | 2.20E-04 | | NA | NA | NA |
| DQB1*06:01 | 4.57E-04 | 2.72E-04 | | 2.42E-03 | -1.97E-03 | 2.72E-04 |
| DQB1*06:02 | 1.36E-01 | 1.71E-02 | | 1.23E-01 | 1.29E-02 | 1.71E-02 |
| DQB1*06:03 | 8.76E-02 | 8.51E-03 | | 9.37E-02 | -6.08E-03 | 8.51E-03 |
| DQB1*06:04 | 3.30E-02 | 4.22E-03 | | 3.23E-02 | 7.11E-04 | 4.22E-03 |
| DQB1*06:09 | 1.98E-03 | 8.53E-04 | | 4.85E-03 | -2.86E-03 | 8.53E-04 |
| DRB1*01:01 | 1.81E-01 | 1.34E-02 | | 1.76E-01 | 4.72E-03 | 1.34E-02 |
| DRB1*01:02 | 1.79E-03 | 6.19E-04 | | 2.39E-03 | -6.00E-04 | 6.19E-04 |
| DRB1*01:03 | 8.34E-04 | 1.29E-03 | | 1.75E-03 | -9.13E-04 | 1.29E-03 |
| DRB1*03:01 | 1.00E-01 | 1.31E-02 | | 9.69E-02 | 3.46E-03 | 1.31E-02 |
| DRB1*04:01 | 9.66E-02 | 2.17E-02 | | 7.90E-02 | 1.76E-02 | 2.17E-02 |
| DRB1*04:03 | 6.43E-04 | 3.40E-04 | | 8.03E-03 | -7.39E-03 | 3.40E-04 |
| DRB1*04:04 | 4.54E-02 | 5.58E-03 | | 3.83E-02 | 7.05E-03 | 5.58E-03 |
| DRB1*04:05 | 4.38E-04 | 2.75E-04 | | 8.93E-04 | -4.55E-04 | 2.75E-04 |
| DRB1*04:07 | 1.94E-03 | 1.90E-03 | | 3.04E-03 | -1.09E-03 | 1.90E-03 |
| DRB1*04:08 | 1.13E-02 | 2.24E-03 | | 1.17E-02 | -4.64E-04 | 2.24E-03 |
| DRB1*07:01 | 5.59E-02 | 6.62E-03 | | 5.88E-02 | -2.86E-03 | 6.62E-03 |
| DRB1*08:01 | 9.65E-02 | 1.06E-02 | | 9.39E-02 | 2.55E-03 | 1.06E-02 |
| DRB1*08:02 | 1.37E-03 | 7.06E-04 | | 4.03E-03 | -2.65E-03 | 7.06E-04 |
| DRB1*08:03 | 9.94E-04 | 6.01E-04 | | 1.18E-03 | -1.84E-04 | 6.01E-04 |
| DRB1*09:01 | 3.57E-02 | 5.28E-03 | | 3.52E-02 | 5.32E-04 | 5.28E-03 |
| DRB1*10:01 | 8.78E-03 | 2.24E-03 | | 9.57E-03 | -7.92E-04 | 2.24E-03 |
| DRB1*11:01 | 3.53E-02 | 4.25E-03 | | 3.13E-02 | 4.00E-03 | 4.25E-03 |
| DRB1*11:03 | 8.94E-04 | 3.96E-04 | | 2.75E-03 | -1.86E-03 | 3.96E-04 |
| DRB1*11:04 | 3.40E-03 | 9.51E-04 | | 6.46E-03 | -3.06E-03 | 9.51E-04 |
| DRB1*12:01 | 2.46E-02 | 4.73E-03 | | 2.63E-02 | -1.72E-03 | 4.73E-03 |
| DRB1*13:01 | 9.55E-02 | 8.94E-03 | | 9.35E-02 | 1.93E-03 | 8.94E-03 |
| DRB1*13:02 | 3.50E-02 | 4.41E-03 | | 3.56E-02 | -6.51E-04 | 4.41E-03 |
| DRB1*13:03 | 4.49E-03 | 1.35E-03 | | 5.66E-03 | -1.17E-03 | 1.35E-03 |
| DRB1*14:01 | 1.94E-03 | 1.04E-03 | | 1.27E-02 | -1.07E-02 | 1.04E-03 |
| DRB1*14:02 | 4.28E-03 | 1.57E-03 | | 4.31E-03 | -3.02E-05 | 1.57E-03 |
| DRB1*14:54 | 9.49E-03 | 1.88E-03 | | 1.14E-04 | 9.37E-03 | 1.88E-03 |
| DRB1*15:01 | 1.38E-01 | 1.70E-02 | | 1.45E-01 | -7.31E-03 | 1.70E-02 |
| DRB1*15:02 | 4.57E-04 | 2.78E-04 | | 1.25E-03 | -7.96E-04 | 2.78E-04 |
| DRB1*16:01 | 8.50E-03 | 2.10E-03 | | 9.08E-03 | -5.82E-04 | 2.10E-03 |

NA, not available; SD, standard deviation.

^a^ The difference (Δ) was calculated by subtracting the imputed allele frequency from the reference allele frequency.

**Supplementary Table 6. Published association data of HLA-alleles and six common autoimmune diseases**

| **Disease** | **Imputed HLA allele** | **Effect size**  **OR (95% CI)** | **P-value** | **Global Biobank Engine^a^ Effect size**  **OR (95% CI)** |
| --- | --- | --- | --- | --- |
| Coeliac disease | DQA1*05:01 | 6.79 (6.27-7.36) | 6.47E-127 | NA |
|  | DQB1*02:01 | 6.80 (6.27-7.36) | 4.58E-127 | 3.95 (3.44-4.54) |
| Type 1 diabetes | DQA1*03:01 | 3.86 (3.55-4.19) | 1.81E-60 | NA |
|  | DQA1*05:01 | 1.64 (1.46-1.83) | 1.05E-05 | NA |
|  | DQB1*02:01 | 1.64 (1.46-1.83) | 9.88E-06 | 2.89 (1.09-7.66) |
|  | DQB1*03:02 | 3.90 (3.60-4.24) | 7.11E-62 | 3.26 (2.63-4.06) |
|  | DRB1*03:01 | 1.64 (1.47-1.84) | 7.99E-06 | NA |
|  | DRB1*04:01 | 3.84 (3.52-4.20) | 4.40E-53 | 2.07 (1.65-2.60) |
| Psoriasis | B*57:01 | 3.00 (2.66-3.38) | 2.46E-20 | 1.55 (1.39-1.74) |
|  | C*06:02 | 2.50 (2.34-2.66) | 8.42E-46 | 2.72 (2.45-3.01) |
| Psoriatic arthritis | B*27:05 | 1.82 (1.62-2.03) | 1.21E-07 | 2.49 (2.1-2.95) |
|  | B*39:01 | 1.60 (1.35-1.89) | 4.93E-03 | 3.49 (2.52-4.85) |
|  | B*57:01 | 3.02 (2.47-3.71) | 5.64E-08 | 1.52 (1.02-2.25) |
|  | C*06:02 | 2.61 (2.34-2.92) | 4.35E-18 | 2.12 (1.78-2.52) |
| Rheumatoid arthritis | DQA1*01:02 | 0.84 (0.79-0.88) | 2.00E-03 | 0.76 (0.72-0.81) |
|  | DRB1*04:01 | 1.14 (1.07-1.23) | 5.73E-02 | 1.38 (1.30-1.46) |
|  | DRB1*04:04 | 1.17 (1.06-1.28) | 1.02E-01 | 1.32 (1.21-1.44) |
|  | DRB1*04:08 | 1.98 (1.70-2.30) | 5.08E-06 | NA |
|  | DRB1*07:01 | 0.90 (0.82-1.00) | 3.01E-01 | 0.75 (0.56-1.00) |
| Multiple sclerosis | DQA1*01:02 | 2.23 (2.10-2.47) | 6.71E-15 | NA |
|  | DQB1*06:02 | 2.64 (2.37-2.93) | 8.86E-20 | NA |
|  | DRB1*15:01 | 2.65 (2.38-2.95) | 3.93E-20 | NA |

CI, confidence interval; NA, not available; OR, odds ratio.

^a^ https://biobankengine.stanford.edu/hla-assoc

# Supplementary Note

# FinnGen

# FinnGen consists of the following people:

# Steering Committee

Aarno Palotie University of Helsinki / FIMM

Mark Daly University of Helsinki / FIMM

## Pharma

Howard Jacob Abbvie

Athena Matakidou Astra Zeneca

Heiko Runz Biogen

Sally John Biogen

Robert Plenge Celgene

Julie Hunkapiller Genentech

Meg Ehm GSK

Dawn Waterworth GSK

Caroline Fox Merck

Anders Malarstig Pfizer

Kathy Klinger Sanofi

Kathy Call Sanofi

## UH & Biobanks

Tomi Mäkelä University of Helsinki / FIMM

Jaakko Kaprio University of Helsinki / FIMM

Petri Virolainen Auria BB / Univ. of Turku /VSSHP

Kari Pulkki Auria BB / Univ. of Turku /VSSHP

Terhi Kilpi THL Biobank (BB) / THL

Markus Perola THL Biobank (BB) / THL

Jukka Partanen Finnish Red Cross Blood Service/FHRB

Anne Pitkäranta HUS/Univ Hosp Districts

Riitta Kaarteenaho Borealis BB/Univ. of Oulu/PPSHP

Seppo Vainio Borealis BB/Univ. of Oulu/PPSHP

Kimmo Savinainen Tampere BB/Univ Tampere/PSHP

Veli-Matti Kosma Eastern Finland BB/UEF/PSSHP

Urho Kujala Central Finland BB /UJy/KSSHP

## Other Experts/ Non-Voting Members

Outi Tuovila Business Finland

Minna Hendolin Business Finland

Raimo Pakkanen Business Finland

# Scientific Committee

## Pharma

Jeff Waring Abbvie

Athena Matakidou Astra Zeneca

Heiko Runz Biogen

Jimmy Liu Biogen

Shameek Biswas Celgene

Julie Hunkapiller Genentech

Dawn Waterworth GSK

Meg Ehm GSK

Josh Hoffman GSK

Dorothee Diogo Merck

Caroline Fox Merck

Anders Malarstig Pfizer

Catherine Marshall Pfizer

Xinli Hu Pfizer

Kathy Call Sanofi

Kathy Klinger Sanofi

## UH & Biobanks

Samuli Ripatti University of Helsinki / FIMM

Johanna Schleutker Auria BB / Univ. of Turku /VSSHP

Markus Perola THL Biobank (BB) / THL

Tiina Wahlfors Finnish Red Cross Blood Service/FHRB

Olli Carpen HUS/Univ Hosp Districts

Johanna Myllyharju Borealis BB/Univ. of Oulu/PPSHP

Johannes Kettunen Borealis BB/Univ. of Oulu/PPSHP

Reijo Laaksonen Tampere BB/Univ Tampere/PSHP

Arto Mannermaa Eastern Finland BB/UEF/PSSHP

Juha Paloneva Central Finland BB /UJy/KSSHP

Urho Kujala Central Finland BB /UJy/KSSHP

## Other Experts/ Non-Voting Members

Outi Tuovila Business Finland

Minna Hendolin Business Finland

Raimo Pakkanen Business Finland

# Clinical Groups

## Neurology Group

Hilkka Soininen LEAD: Kuopio

Valtteri Julkunen Kuopio

Anne Remes Oulu

Reetta Kälviäinen Kuopio

Mikko Hiltunen Kuopio

Jukka Peltola Tampere

Pentti Tienari Helsinki

Juha Rinne Turku

Adam Ziemann AbbVie

Jeffrey Waring AbbVie

Sahar Esmaeeli AbbVie

Nizar Smaoui AbbVie

Anne Lehtonen AbbVie

Susan Eaton Biogen

Heiko Runz Biogen

Sanni Lahdenperä Biogen

Janet van Adelsberg Celgene

Shameek Biswas Celgene

John Michon Genentech

Geoff Kerchner Genentech

Julie Hunkapiller Genentech

Natalie Bowers Genentech

Edmond Teng Genentech

John Eicher Merck

Vinay Mehta Merck

Padhraig Gormley Merck

Kari Linden Pfizer

Christopher Whelan Pfizer

Fanli Xu GSK

David Pulford GSK

## Gastroenterology Group

Martti Färkkilä LEAD: Helsinki

Sampsa Pikkarainen HUS

Airi Jussila Tampere

Timo Blomster Oulu

Mikko Kiviniemi Kuopio

Markku Voutilainen Turku

Bob Georgantas AbbVie

Graham Heap AbbVie

Jeffrey Waring AbbVie

Nizar Smaoui AbbVie

Fedik Rahimov AbbVie

Anne Lehtonen AbbVie

Keith Usiskin Celgene

Tim Lu Genentech

Natalie Bowers Genentech

Danny Oh Genentech

John Michon Genentech

Vinay Mehta Merck

Dermot Reilly Merck

Kirsi Kalpala Pfizer

Melissa Miller Pfizer

Xinli Hu Pfizer

Linda McCarthy GSK

## Rheumatology Group

Kari Eklund LEAD: Helsinki

Antti Palomäki Turku

Pia Isomaki Tampere

Laura Pirilä Turku

Oili Kaipiainen-Seppänen Kuopio

Johanna Huhtakangas Oulu

Bob Georgantas AbbVie

Jeffrey Waring AbbVie

Fedik Rahimov AbbVie

Apinya Lertratanakul AbbVie

Nizar Smaoui AbbVie

Anne Lehtonen AbbVie

David Close AstraZeneca

Marla Hochfeld Celgene

Natalie Bowers Genentech

John Michon Genentech

Dorothee Diogo Merck

Vinay Mehta Merck

Kirsi Kalpala Pfizer

Nan Bing Pfizer

Xinli Hu Pfizer

Jorge Esparza Gordillo GSK

## Pulmonology Group

Tarja Laitinen LEAD: Tampere

Margit Pelkonen Kuopio

Paula Kauppi Helsinki

Hannu Kankaanranta Tampere

Terttu Harju Oulu

Nizar Smaoui AbbVie

David Close AstraZeneca

Susan Eaton Biogen

Steven Greenberg Celgene

Hubert Chen Genentech

Natalie Bowers Genentech

John Michon Genentech

Vinay Mehta Merck

Jo Betts GSK

Soumitra Ghosh GSK

## Cardiometabolic Diseases Group

Veikko Salomaa LEAD: THL

Teemu Niiranen THL

Markus Juonala Turku

Kaj Metsärinne Turku

Mika Kähönen Tampere

Juhani Junttila Oulu

Markku Laakso Kuopio

Jussi Pihlajamäki Kuopio

Juha Sinisalo Helsinki

Marja-Riitta Taskinen Helsinki

Tiinamaija Tuomi Helsinki

Jari Laukkanen Keski-Suomen Keskussairaala

Ben Challis AstraZeneca

Keith Usiskin Celgene

Andrew Peterson Genentech

Julie Hunkapiller Genentech

Natalie Bowers Genentech

John Michon Genentech

Dorothee Diogo Merck

Dermot Reilly Merck

Audrey Chu Merck

Vinay Mehta Merck

Jaakko Parkkinen Pfizer

Melissa Miller Pfizer

Anthony Muslin Sanofi

Dawn Waterworth GSK

## Oncology Group

Heikki Joensuu LEAD: Helsinki

Tuomo Meretoja Helsinki

Olli Carpen Helsinki

Lauri Aaltonen Helsinki

Annika Auranen Tampere

Peeter Karihtala Oulu

Saila Kauppila Oulu

Päivi Auvinen Kuopio

Klaus Elenius Turku

Relja Popovic AbbVie

Jeffrey Waring AbbVie

Bridget Riley-Gillis AbbVie

Anne Lehtonen AbbVie

Athena Matakidou AstraZeneca

Jennifer Schutzman Genentech

Julie Hunkapiller Genentech

Natalie Bowers Genentech

John Michon Genentech

Vinay Mehta Merck

Andrey Loboda Merck

Aparna Chhibber Merck

Heli Lehtonen Pfizer

Stefan McDonough Pfizer

Marika Crohns Sanofi

Diptee Kulkarni GSK

## Opthalmology Group

Kai Kaarniranta LEAD: Kuopio

Joni Turunen HUS/ Secretary

Terhi Ollila HUS

Sanna Seitsonen HUS

Hannu Uusitalo Tampere

Vesa Aaltonen Turku

Hannele Uusitalo-Järvinen PSHP

Marja Luodonpää Oulu

Nina Hautala Oulu

Heiko Runz Biogen

Stephanie Loomis Biogen

Erich Strauss Genentech

Natalie Bowers Genentech

Hao Chen Genentech

John Michon Genentech

Anna Podgornaia Merck

Vinay Mehta Merck

Dorothee Diogo Merck

Joshua Hoffman GSK

## Dermatology Group

Kaisa Tasanen-Määttä Oulu

Laura Huilaja Oulu

Katariina Hannula-Jouppi HUS

Teea Salmi Tampere

Sirkku Peltonen Turku

Leena Koulu Turku

Ilkka Harvima Kuopio

Kirsi Kalpala Pfizer

Ying Wu Pfizer

David Choy Genentech

John Michon Genentech

Nizar Smaoui AbbVie

Fedik Rahimov AbbVie

Anne Lehtonen AbbVie

Dawn Waterworth GSK

# FinnGen Teams

## Admin Team

Anu Jalanko University of Helsinki / FIMM

Risto Kajanne University of Helsinki / FIMM

Ulrike Lyhs University of Helsinki / FIMM

## Analysis Team

Justin Wade Davis Abbvie

Bridget Riley-Gillis Abbvie

Danjuma Quarless Abbvie

Slavé Petrovski Astra Zeneca

Jimmy Liu Biogen

Stephanie Loomis Biogen

Paola Bronson Biogen

Robert Yang Celgene

Joseph Maranville Celgene

Shameek Biswas Celgene

Diana Chang Genentech

Julie Hunkapiller Genentech

Tushar Bhangale Genentech

Natalie Bowers Genentech

Dorothee Diogo Merck

Emily Holzinger Merck

Padhraig Gormley Merck

Xulong Wang Merck

Xing Chen Pfizer

Åsa Hedman Pfizer

Joshua Hoffman GSK

Clarence Wang Sanofi

Ethan Xu Sanofi

Franck Auge Sanofi

Clement Chatelain Sanofi

Mitja Kurki University of Helsinki / FIMM/ Broad Institute

Samuli Ripatti University of Helsinki / FIMM

Mark Daly University of Helsinki / FIMM

Juha Karjalainen University of Helsinki / FIMM/ Broad Institute

Aki Havulinna University of Helsinki / FIMM

Anu Jalanko University of Helsinki / FIMM

Kimmo Palin University of Helsinki / FIMM

Priit Palta University of Helsinki / FIMM

Pietro della Briotta Parolo University of Helsinki / FIMM

Wei Zhou Broad Institute

Susanna Lemmelä University of Helsinki / FIMM

Manuel Rivas University of Stanford

Jarmo Harju University of Helsinki / FIMM

Aarno Palotie University of Helsinki / FIMM

Arto Lehisto University of Helsinki / FIMM

Andrea Ganna University of Helsinki / FIMM

Vincent Llorens University of Helsinki / FIMM

Antti Karlsson Auria BB / Univ. of Turku /VSSHP

Kati Kristiansson THL BB / THL

Mikko Arvas Finnish Red Cross Blood Service BB /FHRB

Kati Hyvärinen Finnish Red Cross Blood Service BB /FHRB

Jarmo Ritari Finnish Red Cross Blood Service BB /FHRB

Tiina Wahlfors Finnish Red Cross Blood Service BB /FHRB

Miika Koskinen Helsinki BB/HUS/Univ Hosp Districts

Olli Carpen Helsinki BB/HUS/Univ Hosp Districts

Johannes Kettunen Borealis BB/Univ. of Oulu/PPSHP

Katri Pylkäs Borealis BB/Univ. of Oulu/PPSHP

Marita Kalaoja Borealis BB/Univ. of Oulu/PPSHP

Minna Karjalainen Borealis BB/Univ. of Oulu/PPSHP

Tuomo Mantere Borealis BB/Univ. of Oulu/PPSHP

Eeva Kangasniemi Tampere BB/Univ Tampere/PSHP

Sami Heikkinen Eastern Finland BB/UEF/PSSHP

Arto Mannermaa Eastern Finland BB/UEF/PSSHP

Eija Laakkonen Central Finland BB /UJy/KSSHP

Juha Kononen Central Finland BB /UJy/KSSHP

## Sample Collection Coordination

Anu Loukola Helsinki BB/HUS/Univ Hosp Districts

## Sample Logistics

Päivi Laiho THL BB / THL

Tuuli Sistonen THL BB / THL

Essi Kaiharju THL BB / THL

Markku Laukkanen THL BB / THL

Elina Järvensivu THL BB / THL

Sini Lähteenmäki THL BB / THL

Lotta Männikkö THL BB / THL

Regis Wong THL BB / THL

## Registry Data Operations

Kati Kristiansson THL BB / THL

Hannele Mattsson THL BB / THL

Susanna Lemmelä University of Helsinki / FIMM

Tero Hiekkalinna THL BB / THL

Manuel Jimenez Gonzalez THL BB / THL

## Genotyping

Kati Donner University of Helsinki / FIMM

## Sequencing Informatics

Priit Palta University of Helsinki / FIMM

Kalle Pärn University of Helsinki / FIMM

Javier Nunez-Fontarnau University of Helsinki / FIMM

## Data Management and IT Infrastructure

Jarmo Harju University of Helsinki / FIMM

Elina Kilpelainen University of Helsinki / FIMM

Timo S Sipilä University of Helsinki / FIMM

Georg Brein University of Helsinki / FIMM

Alexander Dada University of Helsinki / FIMM

Ghazal Awaisa University of Helsinki / FIMM

Anastasia Shcherban University of Helsinki / FIMM

Tuomas Sipilä University of Helsinki / FIMM

## Clinical Endpoint Development

Hannele Laivuori University of Helsinki / FIMM

Aki Havulinna University of Helsinki / FIMM

Susanna Lemmelä University of Helsinki / FIMM

Tuomo Kiskinen University of Helsinki / FIMM

## Trajectory Team

Tarja Laitinen University of Tampere

Harri Siirtola University of Tampere

Javier Garcia University of Tampere

## Biobank Directors

Lila Kallio Auria Biobank

Sirpa Soini THL Biobank

Jukka Partanen Blood Service Biobank

Kimmo Pitkänen Helsinki Biobank

Seppo Vainio Northern Finland Biobank Borealis

Kimmo Savinainen Tampere Biobank

Veli-Matti Kosma Biobank of Eastern Finland

Teijo Kuopio Central Finland Biobank


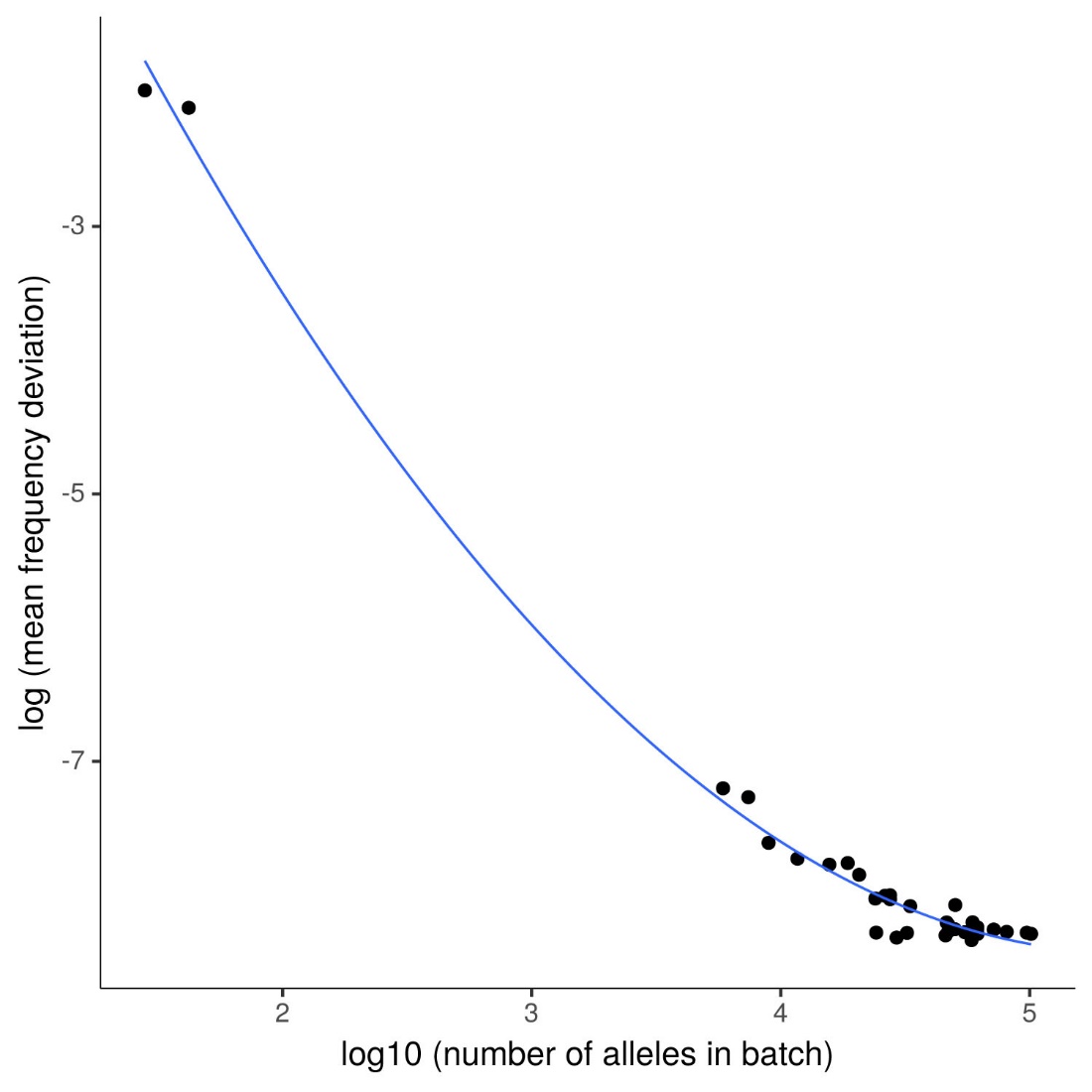


**Supplementary Figure 1. The mean allele frequency deviation vs. the number of imputed alleles in FinnGen R2 genotyping batches.**


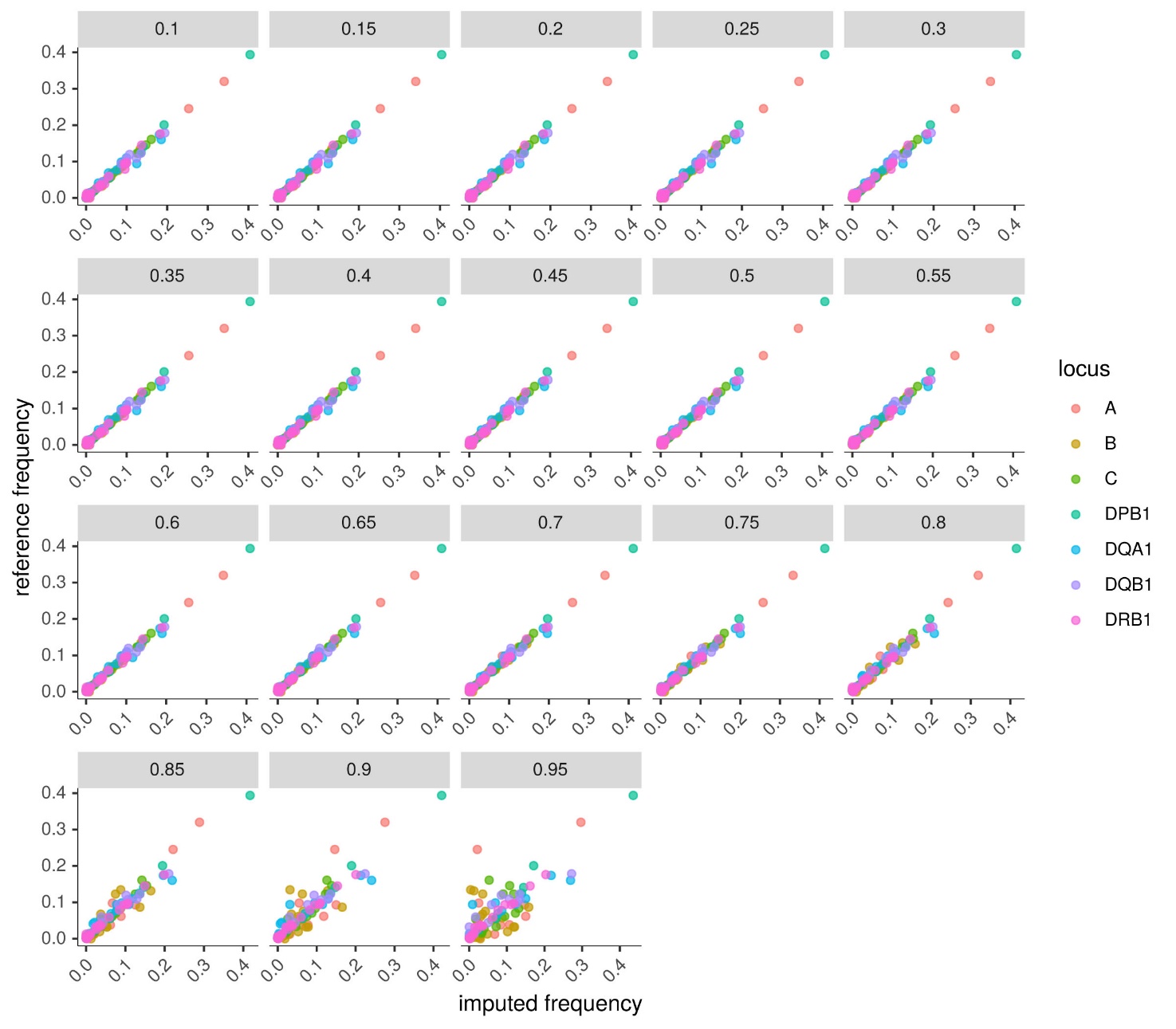


**Supplementary Figure 2. HLA allele frequencies at varying posterior probability cut-offs.** The posterior probability cut-offs are presented in the grey boxes. X-axis, imputed HLA allele frequency; Y-axis, reference HLA allele frequency.

**
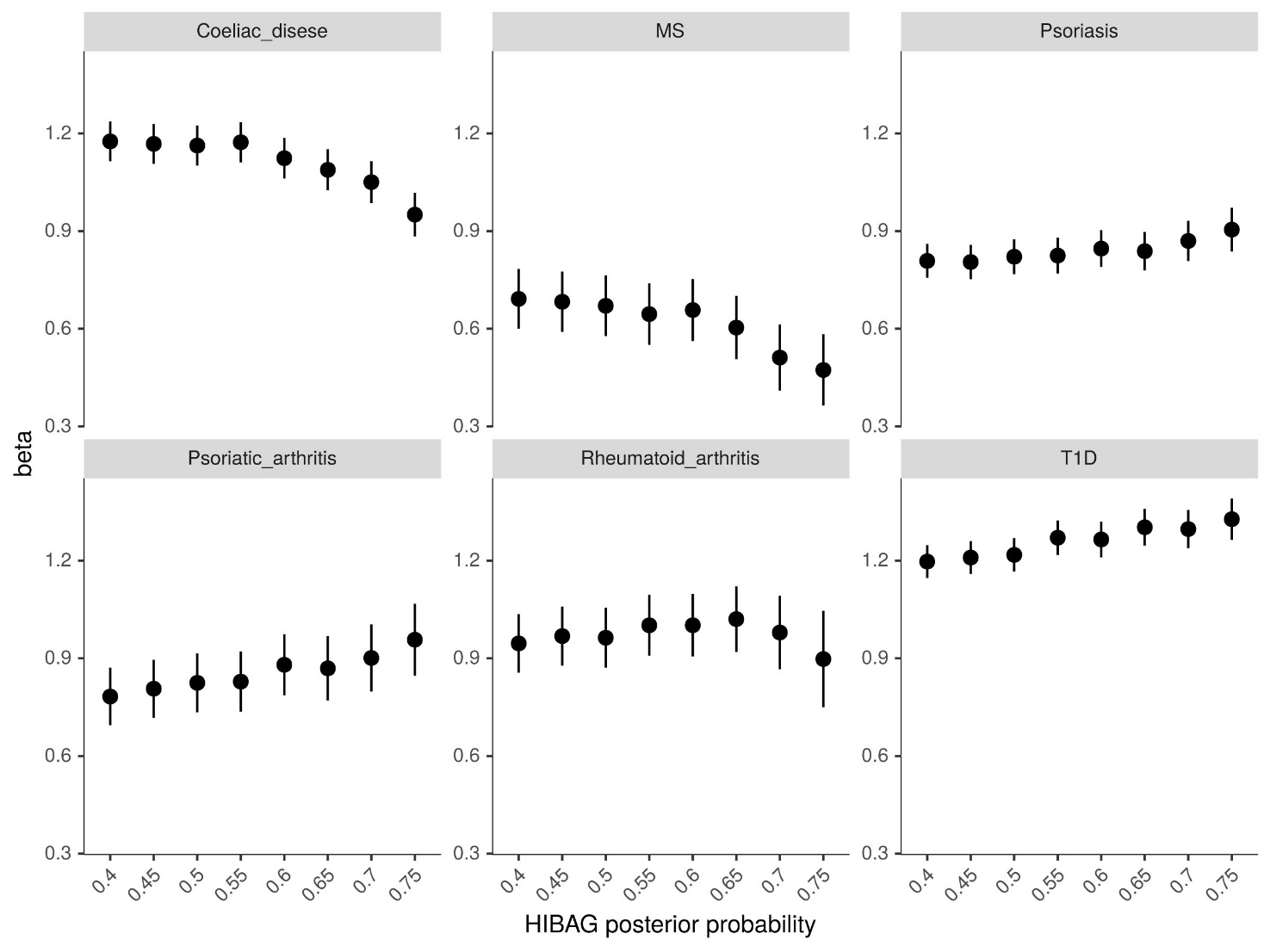
**

**Supplementary Figure 3. Association effect sizes at varying posterior probability cut-offs.** Each panel represents an autoimmune disorder association test (Figure 5 in the main text) at varying HIBAG posterior probability cut-offs for the observed main risk HLA allele.
